# In vivo electrophysiological recordings of the effects of antidepressant drugs

**DOI:** 10.1007/s00221-019-05556-5

**Published:** 2019-05-11

**Authors:** Paul J. Fitzgerald, Brendon O. Watson

**Affiliations:** Department of Psychiatry, University of Michigan, Ann Arbor, MI 48109-5720 USA

**Keywords:** MAOi, SSRI, SNRI, Tricyclic, Ketamine, Gamma oscillations

## Abstract

Antidepressant drugs are a standard biological treatment for various neuropsychiatric disorders, yet relatively little is known about their electrophysiologic and synaptic effects on mood systems that set moment-to-moment emotional tone. In vivo electrical recording of local field potentials (LFPs) and single neuron spiking has been crucial for elucidating important details of neural processing and control in many other systems, and yet electrical approaches have not been broadly applied to the actions of antidepressants on mood-related circuits. Here we review the literature encompassing electrophysiologic effects of antidepressants in animals, including studies that examine older drugs, and extending to more recently synthesized novel compounds, as well as rapidly acting antidepressants. The existing studies on neuromodulator-based drugs have focused on recording in the brainstem nuclei, with much less known about their effects on prefrontal or sensory cortex. Studies on neuromodulatory drugs have moreover focused on single unit firing patterns with less emphasis on LFPs, whereas the rapidly acting antidepressant literature shows the opposite trend. In a synthesis of this information, we hypothesize that all classes of antidepressants could have common final effects on limbic circuitry. Whereas NMDA receptor blockade may induce a high powered gamma oscillatory state via direct and fast alteration of glutamatergic systems in mood-related circuits, neuromodulatory antidepressants may induce similar effects over slower timescales, corresponding with the timecourse of response in patients, while resetting synaptic excitatory versus inhibitory signaling to a normal level. Thus, gamma signaling may provide a biomarker (or “neural readout”) of the therapeutic effects of all classes of antidepressants.

## Introduction

In vivo electrophysiology has been crucial for elucidating important properties of many neural systems, yet these electrical approaches have not yet been broadly applied to the actions of antidepressants. Antidepressant drugs are a standard treatment in a range of neuropsychiatric disorders, including but not limited to major depression, anxiety disorders, obsessive–compulsive disorder, and post-traumatic stress disorder (Cassano et al. 2002; Locher et al. 2017; Puetz et al. 2015). In spite of their widespread use and clinical importance, relatively little is known about their in vivo electrophysiological effects on the brain. Here we focus on the importance of electrophysiologic studies of the neurobiology of these medications and make the argument that increased electrophysiologic study of animals responding to antidepressants may deepen our understanding of both these drugs and limbic circuitry more generally. We also seek to communicate both what has been learned already and to identify open questions that may be addressable with electrophysiologic techniques. Given that the moment-to-moment inner experience of animals and humans is shaped by moment-to-moment neural dynamics best measured electrophysiologically, it is almost certainly necessary to understand electrophysiologic signaling to understand mood and mood disorders. Studying changes due to efficacious antidepressants provides an important inroad into understanding these mood dynamics and brain circuits relating to mood.

Electrophysiologic recordings in animal models have indeed informed our basic understanding of neuronal signaling and brain function, ranging from action potential generation (Hodgkin and Huxley 1952) and synaptic function (Adrian 1914) to later descriptions of visual processing (Hubel and Wiesel 1968), place encoding (O’Keefe and Black 1977) and fear-related circuitry (Milad and Quirk 2002). While as a field we use our understanding of electrophysiologic processing to understand many brain functions in general, we lack a clear conception of how electrophysiologic and usually fast time-scale systems can impact mood and psychiatric disease. More recent advances in systems neuroscience and brain electrophysiology include the additions of the importance of larger coordinated assemblies of neurons (Buzsáki 2010) and the ability of neural oscillations to coordinate both local and long-distance groups of neurons to more precisely control flow of information (Gray and Singer 1989). Little if any such information is commonly used in our understanding of how antidepressants might influence mood- and anxiety-related circuits.

In vivo recording of local field potentials (LFPs), which are voltage waves in the brain that correspond closely with electroencephalographic (EEG) signals, and single neuron action potential firing patterns (spiking) is indeed an effective tool for elucidating circuit-specific neural mechanisms through which antidepressants and other centrally acting drugs produce their therapeutic effects (Blier and El Mansari 2013; Dzirasa et al. 2010; El Mansari et al. 2010; Nagy et al. 2016). When combined with other techniques such as in vivo microdialysis and sophisticated behavioral assays, in vivo electrophysiology can help shed light on systems-level psychopharmacological mechanisms of action and even possibly the physiological basis of major depression itself (Andersson et al. 1995; Belujon et al. 2016; Engin et al. 2008; Linnér et al. 2004).

A perhaps underappreciated aspect of antidepressant drug research is that it provides basic information on the interaction of monoaminergic subcortical nuclei, including the dorsal raphe [which contains serotonin (5-hydroxytryptamine; 5-HT) neurons], locus coeruleus [norepinephrine (NE) neurons], and ventral tegmental area (VTA) and substantia nigra [dopamine (DA) neurons] (El Mansari et al. 2010; Guiard et al. 2008). Perhaps every drug reviewed below, including ketamine, interacts either directly or indirectly with monoaminergic signaling, although this may not constitute the principal therapeutic mechanism of action for each drug. Independent of the effects of a given drug, understanding the functional interaction of these nuclei with each other and with other brain regions may be essential for understanding the basis of mood states and other neural phenomena such as alertness and sleep regulation (Espana and Scammell 2011; Monti and Monti 2007; Samuels and Szabadi 2008).

In this paper we review the antidepressant electrophysiological recording literature in rodents and non-human primates, including studies that examine the following groups of drugs: selective serotonin reuptake inhibitors (SSRIs), serotonin–norepinephrine reuptake inhibitors (SNRIs), tricyclic antidepressants (TCAs), monoamine oxidase inhibitors (MAOIs), norepinephrine–dopamine reuptake inhibitors (NDRIs), miscellaneous antidepressants, novel compounds and potential antidepressants, and NMDA receptor (NMDAR) antagonists. Throughout the review, we describe how these recording data synergize with other techniques, such as in vivo microdialysis and behavioral assays, since these additional techniques were also often used in these studies. As a general rule, the existing studies on monoaminergic drugs appear to have focused on recording in the serotonergic, noradrenergic, and dopaminergic brainstem nuclei, with much less known about their effects on prefrontal or sensory cortex. A number of the studies include data from subregions of hippocampus, whereas there appears to be less known about how antidepressants affect amygdala electrophysiology (Beique et al. 1998; Crespi 2010; Mnie-Filali et al. 2006) (Marcinkiewcz et al. 2016). It is also worth noting that most of these recording studies were carried out in anesthetized animals, with a scarcity of awake (and freely moving) experiments. Another theme is that studies on monoaminergic drugs have tended to focus on single unit firing patterns with less emphasis on LFPs, whereas the NMDAR antagonist literature shows the opposite trend. The latter trend may be due to interest in the effects of NMDAR compounds on brain oscillations in animal models of schizophrenia (Hunt et al. 2015; Kiss et al. 2011a; Lee et al. 2017). Unless noted otherwise, the experiments described below refer to rat anesthetized preparations using systemically administered drugs.

## Literature search details

We conducted a literature search, completed on 7 October 2017, of the Pubmed database. We used the following groups of keywords, where quotation marks were used to group an exact phrase of certain terms: (1) “Single + unit/neuron/cell + recording(s)” + antidepressant(s)/(see LIST below). (2) “Single + unit/neuron/cell + electrophysiology/electrophysiological” + antidepressant(s)/LIST. (3) “In vivo/awake + recording(s)” + antidepressant(s)/LIST. (4) “In vivo/awake + electrophysiology/electrophysiological” + antidepressant(s)/LIST. (5) “local field potential(s)” + antidepressant(s)/LIST. This LIST comprised the following terms (appended to the above searches, one term at a time): escitalopram, fluoxetine, citalopram, sertraline, paroxetine, fluvoxamine, bupropion, reboxetine, duloxetine, venlafaxine, desvenlafaxine, desipramine, nortriptyline, imipramine, clomipramine, amitriptyline, tricyclic, monoamine oxidase inhibitor, MAO inhibitor, tranylcypromine, phenelzine, moclobemide, selegiline, SSRI, SNRI, mirtazapine, ketamine, MK-801. These searches had some degree of redundancy and overlap, to minimize the chances of missing relevant hits. We included all relevant hits in this review.

## Selective serotonin reuptake inhibitors (SSRIs)

A number of in vivo recording studies in the presence of SSRIs show that these drugs acutely dampen firing of neurons in the dorsal raphe nucleus, a primary site of origin of serotonergic forebrain projection neurons (El Mansari et al. 2005; Hajos et al. 1995; Mnie-Filali et al. 2006). This phenomenon may relate to their timecourse of therapeutic efficacy, including their delayed effects in human subjects. A number of these studies examined whether an additional therapeutic treatment, such as physical exercise or another drug, modulated dampened firing. For example, 3 weeks of prior voluntary wheel running in rats counteracted raphe suppression in the presence of acute escitalopram (Dremencov et al. 2017). A number of drugs did in fact counter the SSRI-related reduction in raphe firing including R-citalopram (El Mansari et al. 2007), the potassium channel blocker apamin (Crespi 2010), the selective 5-HT2C receptor antagonist SB242084 (Sotty et al. 2009) as well as 5-HT1A antagonists WAY 100635 (Romero et al. 2003) and (S)-UH-301 (Arborelius et al. 1995), with the latter drug also increasing extracellular 5-HT (Arborelius et al. 1996). Correspondingly an agonist of the same 5-HT1A receptor system, 8-OH-DPAT, decreased dorsal raphe firing more than SSRIs alone (Hajos et al. 1995), as did the DA D2/5-HT2A antagonist risperidone (Dremencov et al. 2007a). These studies collectively suggest that a number of signaling pathways modulate raphe firing, and in particular 5-HT1A receptor-based drugs may, at least in part, alter such firing by interacting with 5-HT1A somatodendritic autoreceptors, although other receptors and circuit targets may also contribute since these drugs were systemically administered. In contrast to some models of SSRI action, drugs that counteract dorsal raphe suppression by SSRIs may actually accelerate the chronic antidepressant properties of the latter drugs, as further described below. Marcinkiewcz et al. (2016) carried out in vivo recordings in the bed nucleus of the stria terminalis (BNST) of mice and found that alterations in single neuron firing during fear acquisition versus recall are present in this brain region, where modulation of a serotonergic inhibitory microcircuit may underlie the initially aversive states induced by exposure to SSRIs such as fluoxetine (Marcinkiewcz et al. 2016).

Chronic administration of SSRIs often produces effects on dorsal raphe firing that differ from those of acute administration (Christensen et al. 2012), a finding that may relate to the acute (side-effect laden) versus chronic (therapeutic) effects of SSRIs in patients. For example, after 2-week treatment with escitalopram in rats, or 3-week treatment using citalopram, the initial suppression of raphe firing had disappeared (El Mansari et al. 2005). This is possibly due to desensitization of somatodendritic 5-HT1A autoreceptors, as the 5-HT1A antagonist (S)-UH-301 acutely increased firing in citalopram-treated animals only, suggesting alterations in receptor sensitization (Arborelius et al. 1995). A related microdialysis study found that, in freely moving rats, chronic citalopram increased extracellular 5-HT in frontal cortex, and this effect was augmented by acute (S)-UH-301 (Arborelius et al. 1996). These studies indicate that SSRIs tend not to depress raphe firing when administered long-term, but they may re-tune 5-HT1A-based regulation which may in part underlie their therapeutic properties.

In human subjects, SSRIs and other monoaminergic antidepressants often require a week or several weeks to begin having a therapeutic effect on major depression (Parker et al. 2000). While these drugs can acutely alter processing of facial expressions of emotion in humans, they typically do not have beneficial effects on mood at this early timepoint (Harmer et al. 2003, 2004), and are even in some patients associated with aversive emotional states upon initial administration (Marcinkiewcz et al. 2016). In rodents, it is well established that SSRIs and related drugs have acute therapeutic effects in the depression-related forced swim test, which suggests a faster timecourse than in humans in some species of animals (Fitzgerald 2009), although Dulawa and Hen have created the novelty-induced hypophagia test for rodents which has been reported to only be sensitive to chronic antidepressant treatment (Dulawa and Hen 2005). Gaining a greater understanding of the electrophysiological correlates of antidepressant response, which span acute to chronic administration and mirror differences in behavioral effect at these timepoints, is an important future challenge for neuroscience.

Other studies of systemically administered SSRIs, show that they interact with medial prefrontal cortex (mPFC) signaling. Acute treatment with fluoxetine in wild type mice did not affect mPFC firing, but did increase spike rates in 5-HT2A knockout mice, where these across-group differences may correspond to poorer novel object recognition in the wild type mice when given this drug (Castañé et al. 2015). Constitutive knockout of this receptor in mice is known to reduce inhibition in conflict anxiety paradigms while having no effect on depression-related behavior (Weisstaub et al. 2006). Three-week, but not acute, treatment with fluoxetine suppressed mPFC cortical firing, which was counteracted by administration of the 5-HT2A (and DA D2) antagonist olanzapine during the last 5 days (Gronier and Rasmussen 2003). Chronic (3 weeks) administration of fluoxetine to 129S1/SvImJ mice, which exhibit deficient fear extinction, yielded reduction of conditioned auditory tone-evoked ventromedial prefrontal cortex firing during fear extinction (Fitzgerald et al. 2014). This drug treatment also suppressed fear during retrieval of extinction. These three studies show that chronic fluoxetine can suppress (or under some circumstances, activate) mPFC signaling, and highlight the role of the 5-HT2A receptor in mPFC regulation.

Experiments using SSRIs also reveal interactions between serotonergic and noradrenergic signaling. Perinatal protein deprivation in rats enhanced locus coeruleus and possibly dorsal raphe activity, and repeated (5 days) fluoxetine treatment normalized this elevated locus coeruleus firing (Sodero et al. 2004). In experimentally naive animals, subacute or chronic (2 weeks) administration of escitalopram suppressed locus coeruleus firing, while co-administration of the D2/5-HT2A antagonist risperidone produced an increase in firing, which the authors attributed to risperidone’s 5-HT2A blocking based on follow-up experiments (Dremencov et al. 2007b). The atypical antipsychotic olanzapine, which also has a 5-HT2A blocking profile, induced an elevation of firing and burst activity in the locus coeruleus, and separate chronic (3 weeks; but not acute) administration of fluoxetine decreased firing and bursting in this nucleus. However, when the two drugs were administered together fluoxetine potentiated the ability of olanzapine to increase locus coeruleus firing and bursting, suggesting a mechanism for the clinical benefits of this drug combination in major depression (Seager et al. 2004). A follow-up study using chronically (3 weeks) administered olanzapine, also found that this drug combination enhanced locus coeruleus activity (Seager et al. 2005). These experiments suggest that SSRIs administered alone can suppress locus coeruleus firing, while paradoxically synergizing with 5-HT2A blocking agents, when used in combination, to elevate firing in this nucleus.

SSRIs also appear to play a role in regulating dopaminergic signaling. One possibility is that when antidepressants boost dopaminergic firing or burst activity it is therapeutic, but the data on SSRIs are ambiguous. For example, chronic (3 weeks) administration of SSRIs (fluoxetine, citalopram, paroxetine) to rats enhanced the firing rates of spontaneously active neurons in the ventral tegmental area (VTA), a major dopaminergic nucleus. Acute injection of 1 mg/kg paroxetine or fluoxetine (2.5 or 5 mg/kg) also increased the number of spontaneously active VTA cells, and an acute injection of 2.5 mg/kg fluoxetine increased firing in substantia nigra pars compacta neurons as well (Sekine et al. 2007). In contrast, chronic (2 weeks) administration of the SSRI escitalopram decreased firing rate and bursting in VTA neurons, whereas citalopram did not affect the overall rate of firing, but did inhibit burst activity. The authors suggest that instances where SSRIs decrease firing in the VTA, an important nucleus in motivation and reward, might lead to lack of an antidepressant response clinically (Dremencov et al. 2009). A third group of researchers found that acute escitalopram increased both the firing rate and bursting activity of VTA neurons, whereas citalopram only increased bursting at a high dosage. They also found that infusing escitalopram, but not citalopram, potentiated NMDA-induced currents in mPFC pyramidal neurons (Schilström et al. 2011). One possibility, suggested in these studies, is that when SSRIs boost VTA signaling in subjects with depression, this helps mediate the antidepressant response, and at least in part distinguishes responders from non-responders.

## Serotonin–norepinephrine reuptake inhibitors (SNRIs)

A series of recording studies have been carried out by Blier and colleagues in rats using two SNRIs, venlafaxine or duloxetine, which are thought to boost both synaptic 5-HT and NE. Beique et al. (1998) compared the ability of acute venlafaxine to suppress firing of dorsal hippocampus CA3 pyramidal cells, versus the SSRI paroxetine or the selective NE boosting agent desipramine, and concluded that venlafaxine is more potent at boosting 5-HT than NE (Beique et al. 1998). Béïque et al. (1999) then compared the effects of venlafaxine as an inhibitor of dorsal raphe and presumably serotonergic firing (referenced against paroxetine), with the effects of venlafaxine as an inhibitor of locus coeruleus and presumably noradrenergic firing (referenced against desipramine), and also concluded that venlafaxine more potently boosts 5-HT than NE (Béïque et al. 1999). To further extend these findings, Béïque et al. (2000a) studied the potency of different doses of chronic (3 weeks) treatment of venlafaxine to counteract the suppression of firing of dorsal hippocampus CA3 pyramidal neurons induced by microiontophoretic application of 5-HT or NE, and found evidence for greater uptake blocking of 5-HT than NE (Béïque et al. 2000a). In separate experiments, they also investigated the ability of acute versus chronic venlafaxine, at different doses, to suppress dorsal raphe or locus coeruleus firing, and found greater sensitivity for blocking 5-HT uptake. Overall, from the above three electrophysiological studies, Blier and colleagues concluded that venlafaxine is indeed more potent at blocking 5-HT than NE synaptic uptake.

A separate study by this group also found that the ability of acute venlafaxine to suppress dorsal hippocampal CA3 pyramidal firing was increased by the beta-adrenergic blocker pindolol, and that the suppression of dorsal raphe firing induced by venlafaxine was counteracted by this agent. This suggests that pindolol may accelerate the antidepressant properties of venlafaxine (Béïque et al. 2000b), possibly by increasing serotonergic transmission. Using similar experiments to the Béïque et al. venlafaxine studies, Blier and colleagues showed that the SNRI duloxetine also exhibits stronger 5-HT than NE boosting properties (Kasamo et al. 1996; Rueter et al. 1998).

## Tricyclic antidepressants (TCAs)

TCAs can block either the 5-HT or the NE reuptake transporter, or both, and they also show some direct antagonistic effects at 5-HT and various other neuromodulator receptors (Pratt et al. 2017). TCAs, like the SSRIs and SNRIs, can acutely suppress firing in either the dorsal raphe or locus coeruleus (or both), depending on the particular drug. This may be expected, given their reuptake blocking profile. For example, subacute treatment with imipramine or acute desipramine in rats suppresses locus coeruleus firing (Linnér et al. 1999; Mcmillen et al. 1980), whereas acute clomipramine suppresses dorsal raphe firing (Gallager and Aghajanian 1975). This acute suppression of serotonergic and/or noradrenergic neural firing tends to decrease upon chronic administration of these drugs, probably at least in part due to desensitization or downregulation of inhibitory somatodendritic autoreceptors (Linnér et al. 1999; Mcmillen et al. 1980; Scuvée-Moreau and Svensson 1982; Svensson and Usdin 1978).

TCAs also influence prefrontal neural activity. For example, recordings in the rat show that acute desipramine increases firing in a subset of prefrontal neurons (Gronier 2011). This drug, however, did not alter firing in VTA or substantia nigra dopaminergic neurons when administered acutely (Chiodo and Bunney 1983). In adult rats that had undergone the stress of undernourishment at a perinatal age, chronic (1 week) desipramine treatment normalized the elevated locus coeruleus activity found in these animals (Nasif et al. 2001). Another experiment that assayed the ability of noradrenergic drugs to modulate the suppressant effect of electrical stimulation of the ascending 5-HT pathway on pyramidal cell firing in the CA3 region of dorsal hippocampus, found that acute desipramine counteracted signaling in this pathway, whereas the NE-lowering drug clonidine at low doses enhanced the pathway (Mongeau et al. 1993). As the authors point out, this last study reinforces the hypothesis that NE can functionally oppose serotonergic signaling. It has also been noted that desipramine can weakly decrease globus pallidus firing rates, whereas the SSRI fluoxetine can weakly increase them (Ruskin et al. 2001).

## Monoamine oxidase inhibitors (MAOIs)

Little is known about the in vivo electrophysiological effects of MAOIs, which block the breakdown of neuronal 5-HT, NE, or DA, where the particular monoamines depend on the drug. The MAO-A inhibitor clorgyline and MAO-B inhibitor pargyline have been shown in rats to acutely potentiate the inhibitory effect on locus coeruleus firing produced by beta-phenylethylamine, a sympathomimetic amine (Lundberg et al. 1985). One interpretation of this study is that these two MAOIs are at least in part boosting synaptic NE, since other drugs such as SNRIs and TCAs that boost NE can acutely inhibit locus coeruleus firing through somatodendritic autoreceptor-mediated mechanisms. Another study found that clorgyline and phenelzine (a non-selective MAOI), when administered chronically (3 weeks; but not when administered only 2 days) decreased firing rates and bursting activity in the dopaminergic VTA (Chenu et al. 2009). Since the MAO-B inhibitor deprenyl was devoid of these effects, the authors concluded that MAO-A inhibition produced this attenuation of dopaminergic neural activity. The only other relevant study on MAOIs we found showed that the MAO-A inhibitor moclobemide at a lower dose suppressed LFP alpha2 and beta1 frequency oscillations, whereas a higher dose decreased spectral power in all frequencies, in a range of brain regions (Dimpfel 2009).

## Norepinephrine-dopamine reuptake inhibitors (NDRIs)

The antidepressant bupropion, which is thought to act as an NE and DA reuptake inhibitor (NDRI), has been shown to normalize suppressed dorsal raphe firing and increase tonic activation of 5-HT1A receptors in the hippocampus, unlike the SSRI paroxetine (El Mansari et al. 2014). Those findings are consistent with bupropion not having strong (or any) 5-HT reuptake modulating properties. Another study found that bupropion inhibits the firing rates of locus coeruleus neurons more effectively than midbrain dopaminergic cells, while not modulating serotonergic dorsal raphe firing (Cooper et al. 1994), similar to the findings of Ghanbari et al. (2010a). One interpretation of these two studies is that the NE reuptake blocking properties of bupropion modulate locus coeruleus firing through feedback mechanisms. Chronically administered bupropion, however, increased dorsal raphe firing while initially suppressing locus coeruleus activity followed by recovery of the latter (El Mansari et al. 2008). Amirabadi et al. (2014) found that acute bupropion tended to inhibit putative GABAergic neurons in the VTA, which the authors suggest may contribute to its clinical side effects (Amirabadi et al. 2014). An additional study using chronic administration of bupropion to study food intake found that this drug modulated nucleus accumbens shell firing rates, and significantly enhanced beta, delta, and theta LFP power (Kalyanasundar et al. 2015).

## Miscellaneous antidepressants

Recording studies have also been conducted using a number of additional antidepressants, whose mechanisms of action may vary widely. For example, the therapeutic mechanism of action of the antidepressant tianeptine is unclear; it may be an enhancer of 5-HT uptake or instead act through glutamatergic modulation, for example (McEwen et al. 2010; Mennini et al. 1987). A study of tianeptine found that it increases AMPA receptor mediated neuronal responses in vivo, and enhances the GluA1-dependent initial phase of long-term potentiation, suggesting its therapeutic effects are produced by facilitating glutamatergic signaling (Szegedi et al. 2011).

The antidepressant trazodone shows electrophysiologic evidence of modulating serotonergic output, and has 5-HT2A/2C receptor blocking properties (Balsara et al. 2005). Two-day treatment with this drug suppressed rat dorsal raphe firing, which recovered to baseline after 2-week treatment. Based on these and further findings of interaction with 5-HT1A signaling and 5-HT levels in the hippocampus, it was suggested that trazodone achieves its therapeutic effects through 5-HT reuptake inhibition and activation of 5-HT1A postsynaptic receptors (Ghanbari et al. 2010b). Earlier studies of this drug had shown that it acutely increases firing of locus coeruleus neurons, and coupled with previous work showing inhibition of dorsal raphe, suggests modulation of both 5-HT and NE signaling by trazodone (Van der Maelen and Braselton 1990).

Ritanserin shows antagonism across a broad spectrum of receptors with highest affinity for blocking 5-HT2A/2C (Javed et al. 1998), and interestingly it has been shown to modulate dopaminergic signaling in the VTA, as well as in the substantia nigra. Andersson et al. (1995) showed that this drug acutely enhanced the firing rate and bursting activity of these two dopaminergic nuclei, and also increased DA concentrations in the mPFC and dorsolateral striatum (Andersson et al. 1995), which may contribute to its mood elevating properties. Ugedo et al. (1989) had observed similar effects on firing and bursting, which were counteracted by the 5-HT depleting drug PCPA, suggesting that 5-HT tonically suppresses these dopaminergic nuclei through 5-HT2 receptors and that ritanserin blocks this effect through its 5-HT2 receptor antagonistic properties (Ugedo et al. 1989). Di Giovanni et al. (1999), however, did not observe basal firing changes in the two nuclei after acute ritanserin, nor did they observe changes in DA levels in the nucleus accumbens or striatum (Di Giovanni et al. 1999). Several studies have also examined the ability of ritanserin to modulate mPFC activity. Ashby et al. (1992) found that microiontophoretic application of 5-HT to this brain region suppressed its firing, an effect that was enhanced by ritanserin (Ashby et al. 1992). This suggests that the 5-HT2A/2C receptors that ritanserin blocks may be functionally opposed to other 5-HT receptors in mPFC. Bergqvist et al. (1999) found that acute ritanserin counteracts the inhibitory effect of microiontophoretic DOI (a 5-HT2A agonist) and mCPP (a 5-HT2C agonist) on mPFC, but not orbitofrontal cortex, activity (Bergqvist et al. 1999). This finding in mPFC reinforces the hypothesis that ritanserin blocks 5-HT2A as well as 5-HT2C receptors.

The antidepressant mirtazapine, which blocks alpha2, 5-HT2, and 5-HT3 receptors (but agonizes 5-HT1A receptors) (de Boer 1996) appears to differ mechanistically from SSRIs, because short-term or acute treatment has been shown to either not affect (Besson et al. 2000) or increase dorsal raphe firing (Haddjeri et al. 1995; Haddjeri et al. 1998a). Based on these acute effects and its interaction with 5-HT1A-modulated dorsal hippocampal CA3 pyramidal firing, mirtazapine may accelerate and strengthen the therapeutic effect of paroxetine (Besson et al. 2000). Acute mirtazapine can also enhance firing of locus coeruleus neurons, and chronic (3 weeks) administration can also increase firing in this nucleus (Haddjeri et al. 1997, 1998a), and lead to tonic activation of postsynaptic 5-HT receptors in the dorsal hippocampus due to desensitization of alpha2-adrenergic heteroceptors on 5-HT terminals (Haddjeri et al. 1995, 1997, 1998a). Thus, electrophysiological evidence suggests mirtazapine interacts with both serotonergic and noradrenergic signaling.

The NE reuptake inhibitor reboxetine appears to have opposite effects from SSRIs since it acutely enhances dorsal raphe firing as well as mPFC extracellular 5-HT, whereas citalopram reduced mPFC 5-HT (Linnér et al. 2004). Moreover, reboxetine and the NE boosting TCA desipramine both tend to enhance septo-hippocampal theta and gamma oscillations, whereas the SSRI fluvoxamine did not strongly influence these oscillations (Hajós et al. 2003). These two studies reinforce the hypothesis that 5-HT and NE are largely functionally opposed in brain functioning. Linnér et al. (2001) showed that acute reboxetine can also modulate dopaminergic signaling by increasing VTA bursting activity (but not firing rate) and enhancing DA output in mPFC, which could relate to the therapeutic efficacy of this drug (Linnér et al. 2001).

## Novel compounds and potential antidepressants

Electrophysiological data suggest that a number of novel compounds also possess antidepressant properties, and further inquiry would help illuminate their therapeutic potential. An extract of the South African plant, *Sceletium tortuosum*, marketed as Zembrin, has been shown to suppress a wide range of LFP frequencies in rats, especially alpha2 and beta1a oscillations, as well as delta and theta frequencies (Dimpfel et al. 2016). The putative antidepressant vortioxetine, which interacts with a range of 5-HT receptor subtypes and is also a 5-HT reuptake inhibitor, has been shown to potently suppress dorsal raphe firing when given acutely. However, this suppression recovers much faster than for fluoxetine, suggesting a faster timecourse of therapeutic action (Bétry et al. 2013). Its therapeutic effects, moreover, may partially arise from desensitization of the 5-HT1B autoreceptor and an increase in the tonic activation of 5-HT1A receptors on dorsal hippocampal CA3 pyramidal cells (El Mansari et al. 2015). Vortioxetine can also increase the power of theta, alpha, and gamma oscillations in motor cortex (Leiser et al. 2014). F15599 is a novel putative 5-HT1A agonist that acutely inhibited dorsal raphe firing at high doses and increased mPFC pyramidal cell firing at a low dose, while also increasing DA release in mPFC (Lladó-Pelfort et al. 2010). Cericlamine is a novel 5-HT reuptake inhibitor that when administered for 2 weeks, results in functional desensitization of somatodendritic 5-HT1A autoreceptors in the dorsal raphe (Jolas et al. 1994). Hence, these four novel compounds may all interact with monoaminergic signaling.

El Mansari and Blier (2008) studied effects of the novel antidepressant, Wf-516, on dorsal raphe and locus coeruleus firing properties in the presence of other compounds, and concluded that it is both a 5-HT1A (autoreceptor) and 5-HT2A antagonist (El Mansari and Blier 2008). Further experiments, including observed effects on dorsal hippocampal CA3 neurons suggested that Wf-516 is also a 5-HT reuptake blocker, and its combination of properties may produce enhanced effectiveness as an antidepressant. Another putative antidepressant, SB-649915-B, counteracted the inhibitory effect of 8-OH-DPAT on dorsal raphe firing (but did not suppress firing when administered alone) and also strongly increased extracellular 5-HT in the cortex. These data are consistent with the view that this compound is a 5-HT1A autoreceptor antagonist, as well as a 5-HT reuptake inhibitor, that may have faster onset than other SSRIs (Hughes et al. 2007). Another novel compound, flesinoxan, suppressed both firing activity of hippocampal CA3 pyramidal and dorsal raphe neurons when systemically administered, and may be a full agonist at presynaptic and partial agonist at postsynaptic 5-HT1A receptors, leading to potential antidepressant effects (Hadrava et al. 1995).

The putative NE reuptake inhibitor nisoxetine increased the bursting activity of DA neurons (recorded in VTA and substantia nigra), while having little effect on firing rate (Shi et al. 2000). Shirokawa et al. (2003) found that infusion of nisoxetine into frontal cortex to test the effects of the drug on locus coeruleus axon terminals indeed produced inhibition of NE uptake, and this was diminished in aged animals (Shirokawa et al. 2003). One possibility is that this compound modulates dopaminergic signaling indirectly, through direct boosting of synaptic NE.

A number of peptidergic compounds may possess antidepressant properties. Somatostatin is a cyclic polypeptide that has antidepressant-like effects in the forced swim test, and suppresses theta oscillations in anesthetized rats, which is a feature that is common to a variety of anxiolytic drugs (Engin et al. 2008). Another peptidergic molecule, spadin, is a K(+) TREK-1 channel blocker that acutely increases dorsal raphe firing; this effect was eliminated by lesioning the mPFC. It also interacted in its effects with the mGluR2/3 antagonist LY 341495, and is proposed to be a candidate rapidly acting antidepressant, at least in part through glutamatergic means (Moha ou Maati et al. 2016). Neurokinin-1 (also called Substance P) antagonists are another class of peptidergic molecules that may influence 5-HT and NE signaling to produce antidepressant effects. For example, the molecules CP-96,345 and CP-99,994 counteracted the suppressant effect of the alpha2 agonist clonidine on locus coeruleus firing, and lesioning NE neurons with DSP-4 prevented the ability of two-day treatment with CP-96,345 to enhance dorsal raphe firing (Haddjeri and Blier 2008). An earlier study by this group had shown that both short- and long-term treatment with CP-96,345 increased dorsal raphe firing, associated with 5-HT1A autoreceptor desensitization (Haddjeri and Blier 2001). Likewise, the neurokinin-1 antagonist L-760735, studied in guinea pigs, activated dorsal raphe firing but without detectable 5-HT1A desensitization, and also increased metabolic activity in a variety of cortical and subcortical structures (Conley et al. 2002). The above three studies collectively suggest that neurokinin-1 antagonists enhance serotonergic, while also modulating noradrenergic, transmission. Thus, several peptidergic candidate antidepressants may influence monoaminergic signaling.

Harmane is a tobacco component that is thought to be an MAO-A inhibitor, and it suppressed dorsal raphe firing in rats, an effect that was reversed by the 5-HT1A antagonist WAY 100635 (Touiki et al. 2005), suggesting serotonergic boosting. Befloxatone is another putative MAO-A inhibitor that can suppress dorsal raphe firing and, after chronic (3 weeks) treatment, attenuate the suppressant effect of clonidine on raphe firing (Haddjeri et al. 1998b; Touiki et al. 2005). Based on recordings in CA3 of dorsal hippocampus, befloxatone can also interact with pindolol to enhance postsynaptic 5-HT transmission (Haddjeri et al. 1998b). These data are consistent with both harmane and befloxatone boosting synaptic 5-HT through MAO-A inhibition, in addition to other potential neurochemical effects.

Carisbamate is an anticonvulsant that is thought to modulate voltage-gated sodium channels. It can decrease neural firing in dorsal raphe, locus coeruleus, and VTA, but instead increase tonic activation of postsynaptic 5-HT1A receptors in dorsal CA3 pyramidal cells, and this may produce an antidepressant-like effect through serotonergic attenuation of glutamatergic signaling (Shim et al. 2013). Two novel triple (monoamine) reuptake inhibitors, SEP-225289 and DOV216303, acutely inhibited firing in dorsal raphe, locus coeruleus, and VTA, with the strongest inhibition in locus coeruleus (Guiard et al. 2011). This electrophysiological profile is consistent with monoamine reuptake inhibition that is observed more selectively in SSRIs and TCAs. Pindolol is a beta adrenergic-5-HT1A/1B receptor antagonist that may not have intrinsic antidepressant properties (although it can alter dorsal raphe firing) but may enhance the effects of SSRIs via serotonergic modulation (Haddjeri and Blier 2000; Sprouse et al. 2000). The novel antidepressant nomifensine acutely inhibits firing of dopaminergic VTA neurons, so it may produce its therapeutic effects through DA reuptake inhibition (Einhorn et al. 1988). Finally, we were not able to find any in vivo recording data on the mu opioid receptor modulating drug, buprenorphine (Robinson et al. 2017), which is currently undergoing clinical trials for use in major depression (Garay et al. 2017).

## NMDA receptor antagonists

The NMDAR antagonist ketamine has been at the forefront of mood disorder research since its initial demonstration as a rapidly acting antidepressant in human subjects (Berman et al. 2000). While a number of electrophysiological studies have been carried out in rodents in the presence of NMDAR antagonists, where many of these studies were investigating the NMDAR hypofunction model of schizophrenia (Hakami et al. 2009; Hunt et al. 2008, 2015; Kiss et al. 2011a; Lee et al. 2017), most have focused on LFP oscillations (and their relationship with cognition in the disease) rather than single unit activity. So at this point, little is known about single unit firing in the rapidly acting antidepressant ketamine, or the related NMDAR antagonists dizocilpine (MK-801) and phencyclidine (PCP). One finding that has been replicated a number of times in these studies, including for ketamine in vervet monkeys (Slovik et al. 2017) and macaques (Skoblenick et al. 2016), is that acute systemic administration of these three drugs, and in some cases infusion of them into local brain regions, tends to enhance gamma and high frequency oscillations (HFO) in a number of cortical and subcortical structures (Hakami et al. 2009; Hunt et al. 2008, 2009, 2010; Kealy et al. 2017; Kjaerby et al. 2017; Lee et al. 2017; Matulewicz et al. 2010; Nagy et al. 2016; Nicolás et al. 2011; Olszewski et al. 2013; Wood et al. 2012; Hunt et al. 2006; Sullivan et al. 2015; Maheshwari et al. 2016). Of note, Hunt et al. (2015) found that acute MK-801 not only increased the power of nucleus accumbens HFO but also produced a small increase in frequency, whereas the 5-HT2A antagonist clozapine and 5-HT1A agonist 8-OH-DPAT each counteracted this increase in frequency, suggesting functional opposition between these two 5-HT receptor subtypes in this effect (Hunt et al. 2015). In an earlier study, Hunt et al. (2006) had shown that acute ketamine injections not only increase HFO power in the nucleus accumbens but also induce hyperactivity in freely moving rats (Hunt et al. 2006). It has also been suggested that in the mouse, ketamine enhances both background and auditory-evoked gamma power, while attenuating theta oscillations (Lazarewicz et al. 2010). In rats MK-801 has also been shown to decrease the frequency and power of hippocampal theta oscillations (Pitkänen et al. 1995), and has also been associated with increasing power of low frequency (1–6 Hz) and decreasing the power of higher frequency (16–32 Hz) oscillations across widespread cortical and subcortical structures (Ehlers et al. 1992). In mice that were chronically (1 week) given ketamine, this produced a decrease in visually evoked low and high gamma, but a trend toward an increase in baseline power in high gamma (Hamm et al. 2017).

Ketamine/xylazine anesthesia can also alter gamma oscillations, including somatosensory-motor cortical synchrony of these oscillations, reflecting changes in the integration of information across disparate cortical areas (Hwang et al. 2013). An additional study noted that gamma oscillations are enhanced under ketamine versus urethane anesthesia (Sharma et al. 2010).

In terms of thalamo-cortical oscillations in particular, acute MK-801 has been shown in anesthetized rats to transform regular 2 Hz delta oscillations into a less regular 0.5–1.5 Hz delta rhythm (Kiss et al. 2011a). This drug, when infused into the VTA, has also been shown to decrease tail pinch-evoked theta (peak power) while increasing delta peak power (Matulewicz et al. 2014). Acute ketamine can also depress LFPs in orbitofrontal cortex evoked by excitatory thalamic afferent stimulation (Patton et al. 2017), and acute PCP can alter thalamo-cortical oscillations as well, particularly for those below 4 Hz (Troyano-Rodriguez et al. 2014). A series of studies by Artigas and colleagues, in mice and rats, has indeed shown that acute PCP alters single unit firing and disrupts slow (< 4 Hz) oscillations in prefrontal cortex, through modulation of the reticular nucleus (and other nuclei) of the thalamus. These effects can be counteracted by a number of both typical and atypical antipsychotic drugs (Kargieman et al. 2012; Lladó-Pelfort et al. 2016; Santana et al. 2011; Troyano-Rodriguez et al. 2014).

Another topic that has been addressed in a number of NMDAR antagonist studies in rodents comprises the effects of these drugs on dopaminergic signaling, since DA may play a prominent role in the pathophysiology of schizophrenia (Carlsson 2006; Jauhar et al. 2017). Most of these NMDAR antagonist studies have focused on the VTA, as well as the substantia nigra, in anesthetized preparations. Their general conclusion is that ketamine, MK-801, and PCP tend to increase the mean firing rate, as well as bursting activity, in these nuclei (Belujon et al. 2016; Bennett and Gronier 2007; French et al. 1993; Murase et al. 1993; Steinfels et al. 1989; Zhang et al. 1992). An additional study in the basal ganglia found that MK-801 counteracted changes in the firing patterns of caudate and globus pallidus neurons induced by the dopaminergic agonist drug apomorphine (Kelland and Walters 1992). In a study of learned helplessness in Wistar-Kyoto rats, ketamine was able to restore decreased DA population activity, as well as synaptic plasticity in the hippocampus-accumbens pathway, brought about by exposure to inescapable footshock stress (Belujon and Grace 2014).

NMDA receptor antagonists may also alter signaling in the NE-locus coeruleus system. In chloral hydrate anesthetized rats, acute PCP and MK-801 each decreased locus coeruleus firing rates while also decreasing neural responses in this nucleus to electrical stimulation of the hindpaw (i.e., a sensory stimulus) (Murase et al. 1992). However, a study of acute morphine withdrawal in rats found that MK-801 failed to suppress elevated locus coeruleus firing or the observed increased NE turnover in a variety of brain structures, although this drug did suppress behavioral signs of withdrawal (Rasmussen et al. 1991). A more recent study using chloral hydrate anesthesia found that subanesthetic doses of ketamine did not alter mean firing rates in the dorsal raphe or VTA, but did increase firing in the locus coeruleus (El Iskandrani et al. 2015). While these three experiments differ in a number of aspects, one possibility is that their discrepancies in drug effect are related to differences in dose across the three drugs.

Several studies have investigated the effects of NMDAR antagonists on prefrontal signaling, especially mPFC, which is a locus of interest in a range of neuropsychiatric disorders including major depression (Groves et al. 2018). Kiss et al. (2011b) found that, whereas systemic administration of MK-801 to rats produced an overall decrease in mPFC multi-unit activity with diverse effects on single units, microinfusion of MK-801 into this brain region did not have these effects. The authors then concluded that the cortex is not likely to be the primary site of action of systemically administered NMDAR antagonists (Kiss et al. 2011b). In freely moving rats, Molina et al. (2014) found an overall increase in mPFC unit firing and gamma power with MK-801, and the synchronization of firing became more irregular (Molina et al. 2014). Using a combination of recording in freely moving rats and computational modeling, Moran et al. (2015) concluded that NMDAR antagonists (like schizophrenia itself) may disrupt top-down processing from areas like mPFC that ordinarily communicate well with lower order brain structures to facilitate predictive coding. In this scenario, schizophrenia and its associated theta and gamma abnormalities may disrupt prediction error processing, leading to false perceptual inferences (Moran et al. 2015). Another group of researchers, however, found no evidence that NMDAR antagonists disinhibit PFC pyramidal neurons, which has been proposed to underlie psychosis in schizophrenia, but did observe potential disconnections of spike-discharge from gamma oscillations (Wood et al. 2012). In an earlier study by this group, Homayoun and Moghaddam (2007) found that acute treatment with clozapine, but not haloperidol, reversed population increases in mPFC firing produced by MK-801 (Homayoun and Moghaddam 2007). In another study, Labonte et al. (2009) investigated the ability of systemic MK-801 to modulate the effect of microinfusions of 5-HT and NMDA into mPFC, and concluded that MK-801 modifies serotonergic synapses in the mPFC by enhancing excitatory 5-HT2A/2C responses and suppressing NMDA-induced excitation, with potential relevance to pathophysiology in schizophrenia (Labonte et al. 2009).

## Discussion and hypotheses

Here we have reviewed the existing in vivo electrophysiological studies that probed the neural effects of antidepressants in rodents and, to a much more limited degree, in non-human primates. This is a growing literature that is characterized to some degree by what is known, but also by what remains to be adequately addressed (see Table 1 for a summary of firing rate data; Table 2 summarizes LFP findings). As mentioned earlier, these studies have historically emphasized the effects of neuromodulatory (i.e., monoaminergic) drugs on the firing patterns of neurons in dorsal raphe (for 5-HT neurons), locus coeruleus (NE), and substantia nigra and VTA (DA), focused on autoreceptor (as well as heteroceptor) regulation (Blier et al. 1987, 1990). The studies by Blier and colleagues have also extended this concept, particularly for 5-HT, to postsynaptic interaction with the dorsal hippocampus CA3 subregion (Beique et al. 1998; Haddjeri et al. 1997; Mongeau et al. 1993). Most of the antidepressant drugs reviewed above indeed influence monoaminergic signaling, although it is not known if doing so is their only (or principal) mechanism of action. For the NMDAR antagonist drugs, including ketamine and MK-801, most of the studies have focused on LFP oscillations that relate these drugs with the NMDAR hypofunction model of schizophrenia (Coyle et al. 2003; Frohlich and Van Horn 2014; Jadi et al. 2016). What remains neglected in the antidepressant literature as a whole is a detailed understanding of the electrophysiological effects of these drugs on all subregions of neocortex, including prefrontal, motor, and sensory areas. While several of the above studies have addressed mPFC, even less is known about these other areas, including orbitofrontal cortex, which plays an important role in reward-seeking, goal-directed behavior and possibly anhedonia (Furuyashiki et al. 2008; Romer Thomsen et al. 2015; Stalnaker et al. 2015; Zhang et al. 2016).

**Table 1:** Summary of in vivo electrophysiology mean firing rate data

Drug class	Drug	DRN	LC	VTA	Subst nigra	Globus pallidus	HC	mPFC	OFC
SSRIs	Acute escitalopram	↓	↓	↕↔					
	Chronic escitalopram	↓↔	↓	↓			↑		
	Acute citalopram	↓	↔	↔	↔				
	Chronic citalopram	↓↔		↑↔	↔		↑		
	Acute paroxetine	↓	↔	↑	↔		↓		
	Chronic paroxetine	↔		↑	↔				
	Acute fluoxetine	↓	↔	↑↔	↑↔	↑		↔	
	Chronic fluoxetine	↓↔	↓	↑	↔			↓	
SNRIs	Acute venlafaxine	↓	↓				↓		
	Chronic venlafaxine	↔	↓						
	Acute duloxetine	↓	↓						
	Chronic duloxetine	↔							
TCAs	Acute desipramine		↓	↔	↔	↓		↑	
	Chronic desipramine		↓↔	↑	↑				
	Acute imipramine		↓						
	Chronic Imipramine		↓						
	Acute chlomipramine	↓							
MAOIs	Acute clorgyline			↔					
	Chronic clorgyline			↓					
	Acute deprenyl			↔					
	Chronic deprenyl			↔					
	Acute phenelzine			↔					
	Chronic phenelzine			↓					
NDRIs	Acute bupropion	↑↔	↓	↓↔	↓				
	Chronic bupropion	↑	↓↔	↔					
Misc	Acute trazodone	↓	↑						
	Chronic trazodone	↔							
	Acute mirtazapine	↑↔	↑						
	Chronic mirtazapine	↑	↑						
	Acute ritanserin			↑↔	↑↔			↓	
	Acute reboxetine	↑		↔					
Novel	Acute vortioxetine	↓							
	Chronic vortioxetine	↔							
	Acute F15599	↓						↑	
	Acute cericlamine	↓							
	Chronic cericlamine	↔							
	Acute Wf-516	↓							
	Acute SB-649915-B	↔							
	Acute flesinoxan	↓					↓		
	Acute nisoxetine			↑	↑				
	Acute spadin	↑							
	Acute CP-96,345	↑↔	↔						
	Chronic CP-96,345	↑							
	Acute CP-99,994		↔						
	Chronic CP-99,994		↔						
	Acute L-760735	↑							
	Acute harmane	↓							
	Acute befloxatone	↓							
	Chronic befloxatone	↔							
	Acute carisbamate	↓	↔	↔					
	Chronic carisbamate	↓	↓	↓					
	Acute SEP-225289	↓	↓	↓					
	Acute DOV216303	↓	↓	↓					
	Acute Pindolol	↓					↓		
	Acute nomifensine			↓					
NMDAR antag	Acute MK-801		↓	↑	↑			↕↔	↑
	Acute PCP		↓	↑				↑	
	Acute ketamine	↔	↑	↑↔				↑	

Arrows indicate an increase (↑) in neuronal population mean firing rate, decrease (↓), or no change (↔), found in at least one study. Chronic includes 5 days or more; acute includes 1 and 2 day treatments as well as single injections. Table does not include studies where 5-HT or NE was microinfused in dorsal hippocampal CA3 and firing rates were modulated by one of these antidepressants, or other drug interaction studies. Only includes wild type animals and systemically administered drugs. Table does not include LTP/LTD information

*DRN* dorsal raphe nucleus,* LC* locus coeruleus,* VTA* ventral tegmental area,* Subst nigra* substantia nigra,* mPFC* medial prefrontal cortex,* OFC* orbitofrontal cortex,* HC* hippocampus

**Table 2:** Summary of in vivo local field potential (LFP) data

Drug class	Drug	Subst nigra	Amygd	Thalms	Nuc acmb	HC	mPFC	OFC	Mot cortx	Sens cortx
SSRIs	Acute escitalopram								↔Alpha, theta, gamma	
	Acute citalopram									↓Gamma
	Acute fluvoxamine					↔Theta, gamma				
SNRIs	Acute duloxetine								↔Theta, gamma, ↓alpha	
TCAs	Acute desipramine					↑Theta, ↑gamma (trend)				
MAOIs	Acute moclobemide					↓All freq		↓All freq		
NDRIs	Chronic bupropion				↑Beta, delta, theta					
Misc	Acute reboxetine					↑Theta, gamma				
Novel	Acute vortioxetine								↑Theta, alpha, gamma	
	Acute F15599						↔Delta			
	Acute Zembrin					↓Alpha, beta, delta, theta				
NMDAR antag	Acute MK-801		↑Delta, theta, ↔alpha, ↓beta, gamma	↑Delta, ↕theta, ↔alpha, ↓beta, gamma	↑hfo, ↕gamma, ↓delta	↑hfo, ↕gamma, theta, ↔delta, alpha, beta	↑hfo, ↑gamma, disrupts delta	↑Gamma		↑hfo, gamma
	Acute PCP					↔All freq	↓Delta			
	Acute ketamine	↑hfo, gamma			↑hfo, gamma	↑hfo, ↕gamma, ↓theta, ↕delta, ↓alpha	↑Gamma, ↓theta	↓Unspecified freq		↑gamma, delta
	Chronic ketamine									↓gamma

Arrows indicate an increase (↑) in LFP power (averaged across all animals) in that frequency band, decrease (↓), or no change (↔), found in at least one study. Chronic includes 5 days or more; acute includes 1 and 2 day treatments as well as single injections. Table does not include drug interaction studies. Only includes wild type animals and systemically administered drugs. Only invasive, intracranial LFP recordings are shown, not skull surface recordings

Abbreviations (see Table 1 legend as well):* Amygd* amygdala,* Thalms* thalamus,* Nuc acmb* nucleus accumbens,* Mot cortx* motor cortex,* Sens cortx* sensory cortex

**Fig. 1:**
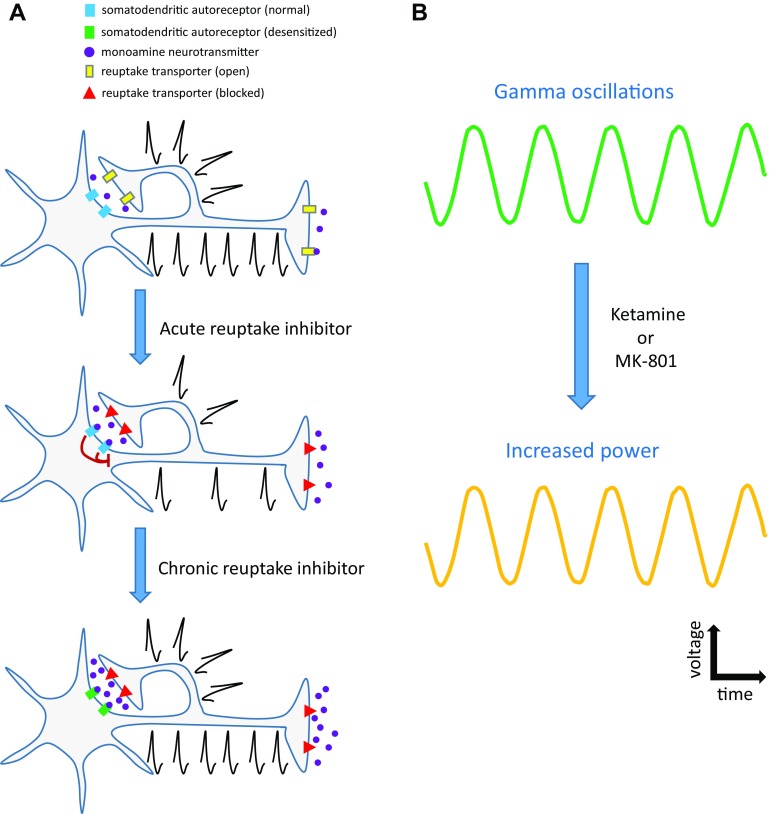
Major conclusions (or working hypotheses) from these studies. **a** Most antidepressants acutely dampen action potential firing in monoaminergic brainstem nuclei due to somatodendritic autoreceptor mediated inhibition, which typically desensitizes upon chronic drug administration. **b** The NMDAR antagonists ketamine and MK-801 acutely enhance the power of gamma and high frequency voltage oscillations in a variety of brain regions

In spite of the missing information at this time, we reach the following two major conclusions (or working hypotheses) regarding these studies: (1) most antidepressants acutely dampen firing in monoaminergic brainstem nuclei due to autoreceptor mediated inhibition, which typically desensitizes upon chronic drug administration; (2) the NMDAR antagonists ketamine and MK-801 acutely enhance gamma and high frequency oscillations. These two hypotheses (illustrated in Fig. 1) have important basic, translational, and clinical ramifications, which we address in greater detail below.

Regarding the first major conclusion: a number of the studies reviewed above addressed the effects of monoaminergic reuptake inhibiting antidepressants (i.e., SSRIs, SNRIs, TCAs) on dorsal raphe, locus coeruleus, and VTA, neural firing patterns. These studies consistently found that acute administration of drugs that boost 5-HT, NE, or DA, respectively, inhibit dorsal raphe, locus coeruleus, and VTA firing (Crespi 2010; Linnér et al. 1999; Svensson and Usdin 1978). Somatodendritic autoreceptors are thought to play a critical role in this process, since they are sensitive to the extracellular concentration of their respective neurotransmitter and inhibit action potential generation in a negative feedback manner (Arborelius et al. 1996; Linnér et al. 1999; Nasif et al. 2001). But since these drugs are administered systemically and are presumably distributed throughout the brain, acute inhibition of firing in monoaminergic nuclei may also be mediated by other circuit elements, including axon terminal autoreceptors that inhibit neurotransmitter release to influence postsynaptic neurons, such as prefrontal neurons that feed back on the brainstem nuclei (Hajos et al. 1999; Sotty et al. 2009). There could also be inhibitory interactions between the monoaminergic nuclei (Guiard et al. 2008; Mongeau et al. 1993; Seager et al. 2004) that modulate these processes. In spite of these caveats, inhibitory somatodendritic autoreceptors appear to play a critical role in this process, and desensitization of them appears to lead to recovery of cell firing upon chronic administration of these drugs (Béïque et al. 2000a; Blier et al. 1987). The timecourse of this process does not necessarily mimic the timecourse of therapeutic response, since rodents exhibit antidepressant-like responses to these drugs within minutes of administration in the forced swim and tail suspension tests (Fujishiro et al. 2001; Leggio et al. 2008), and may still be acutely producing a net increase in transmitter efflux at their axon terminals presumably due to reuptake inhibition (Arborelius et al. 1996; Gallager and Aghajanian 1975). And on that note, a final point is that action potential firing rate does not necessarily equate with the amount of transmitter released at the axon terminal, since other factors influence this, such as the number of synaptic vesicles released per impulse (Kaeser and Regehr 2017).

The data from the first major conclusion may also suggest that 5-HT and NE are functionally opposed in a number of brain circuits, which may be a general principle describing many of their interactions in vivo. A number of studies reviewed above contribute to this hypothesis. For example, release of these two transmitters is regulated in an opposing fashion by the VTA (Guiard et al. 2008). Additionally, antidepressants that either boost 5-HT or NE selectively, have opposing effects on gamma oscillations, with 5-HT suppressing and NE enhancing them (Akhmetshina et al. 2016; Hajós et al. 2003; Mendez et al. 2012). Chronic administration of SSRIs such as escitalopram and fluoxetine suppress locus coeruleus firing, providing direct evidence for the functional opposition hypothesis (Dremencov et al. 2007a, b; Seager et al. 2004). The noradrenergic TCA desipramine attenuates activation of the ascending 5-HT pathway onto CA3 of the dorsal hippocampus, providing further support (Mongeau et al. 1993). The principle of functional opposition may extend to pairs of cortical areas that 5-HT and NE may modulate, such as the prelimbic and infralimbic subdivisions of mPFC, which appear to have opposing effects on conditioned fear responses, as well as on drug seeking behavior (Peters et al. 2009; Vidal-Gonzalez et al. 2006).

A point that is related to 5-HT-NE opposition is that the SNRIs venlafaxine and duloxetine may boost 5-HT more than NE, a conclusion reached by Blier and colleagues after a series of studies on these two compounds, described above (Beique et al. 1998; Béïque et al. 1999; Béïque et al. 2000a; Kasamo et al. 1996; Rueter et al. 1998). If these two drugs are functionally more like SSRIs than like noradrenergically selective TCAs (such as desipramine or nortriptyline), then this may inform decisions on when they should be used in individuals with major depression or other neuropsychiatric disorders, while also having implications for translational research in animal models.

Another point relevant to 5-HT-NE opposition is that the TCA imipramine may boost NE more strongly than 5-HT. This hypothesis is based on the finding above that imipramine acutely suppresses locus coeruleus firing and in this manner resembles the selective NE reuptake blocker, desipramine (Béïque et al. 1999; Linnér et al. 1999; Mcmillen et al. 1980; Svensson and Usdin 1978). Further, it is known that in vivo, imipramine is rapidly metabolized into desipramine (Gram 1988; Strandgarden and Gunnarsson 1994). If imipramine is largely functionally an NE-specific reuptake inhibitor, this would have immediate translational and clinical ramifications, as described above for venlafaxine and duloxetine. Imipramine is indeed still widely used clinically, with a rich history in the literature, whose studies may be interpreted somewhat differently if this drug is principally selective for NE in vivo.

A second and final major conclusion is that the NMDAR antagonist drugs, such as ketamine and MK-801, enhance the power of gamma and high frequency oscillations (HFO) when acutely administered. This was demonstrated in a number of the studies reviewed above, where many of these experiments were testing these drugs in the NMDAR hypofunction model of schizophrenia (Hakami et al. 2009; Hunt et al. 2008; Hunt et al. 2015; Kiss et al. 2011a; Lee et al. 2017) rather than assaying their rapidly acting antidepressant-like effects (Berman et al. 2000; Engin et al. 2009). Other experiments above found enhancement of gamma and/or HFO when using ketamine to maintain anesthesia (Chery et al. 2014; Hakami et al. 2009; Hwang et al. 2013; Sharma et al. 2010). Interestingly, ketamine is also known to dose-dependently boost NE, with the greatest boosting at an anesthetic dose (i.e., 100 mg/kg) in rodents (Kubota et al. 1999a, b). This is paradoxical, given that NE promotes wakefulness from isoflurane anesthesia and natural sleep (Aston-Jones et al. 2001; Vazey and Aston-Jones 2014), although NE may play a yet-to-be-determined role in ketamine’s anesthetic properties, which includes suppression of prefrontal glutamate release at high doses (Moghaddam et al. 1997). For our purposes, NE itself is also known to enhance gamma oscillations in awake animals (Gire and Schoppa 2008; Hajós et al. 2003; Pandipati et al. 2010).

In suggesting that acute administration of NMDAR antagonists increases gamma power in a number of brain regions, we must consider several caveats. One is that chronic NMDAR blocking can actually decrease gamma power in sensory brain regions, for example (Hamm et al. 2017). Also a more refined view of gamma oscillations as a biomarker for major depression must take into consideration that increases in “power” may comprise a mixture of actual oscillatory activity as well as non-synchronized fast synaptic activity (Fitzgerald and Watson 2018). Gamma power may also vary as a function of exact brain region, stimulus type, degree of alertness or anesthetic state, and type or dose of NMDAR antagonist drug (Fitzgerald and Watson 2018) and, therefore, simple generalizations of frequency band power and drug may not be valid—but gamma band power does strongly increase acutely after low dose ketamine across many regions of the forebrain.

Integrating the above electrophysiological data on NMDAR antagonists with the findings on neuromodulator-based drugs, we propose the following common pathway for antidepressant response, partly to form a bridge in the existing gaps in the electrophysiological literature noted above. In this scenario, neuromodulatory antidepressants, and NMDAR antagonists have similar final effects on limbic circuitry such as the anterior cingulate cortex and hippocampus. Whereas NMDAR blockade may induce a high powered gamma oscillatory state via direct and fast alteration of glutamatergic systems in mood-related circuits, neuromodulator-based antidepressants may induce similar effects over slower timescales, corresponding to the timecourse of response in patients. In particular, “optimal” (i.e., not too much or too little) gamma power induced by all therapeutically effective antidepressants corresponds to long-term neural changes that reset synaptic excitatory versus inhibitory signaling to a normal level (Fee et al. 2017; Nugent et al. 2018; Watson et al. 2018). Thus, gamma signaling may provide a biomarker (or “neural readout”) of the therapeutic effects of all classes of antidepressants (Fitzgerald and Watson 2018). We must emphasize, however, that this is a preliminary hypothesis, especially considering the gaps in the literature highlighted throughout this article, both for neuromodulatory and NMDAR antagonist antidepressants with respect to brain region. We are only suggesting here that a unified framework for understanding the therapeutic effects of all classes of antidepressants on neurocircuits must reconcile their very different initial neural effects and mechanisms of action. Another possibility is that different classes of antidepressants do not share the same final effect on neural circuits, and simply achieve their therapeutic effect in different ways.

To summarize, here we have reviewed the literature on in vivo electrophysiologic studies in the presence of antidepressant drugs. While a number of pioneering studies have already been carried out, most prominently by Blier et al., our understanding of the effects of these drugs throughout the brain is not yet well developed. Underdeveloped areas for future inquiry include more recordings in awake, freely moving animals with a focus on neocortical areas, although subcortical structures such as amygdala, hippocampus, thalamus, and the brainstem monoaminergic nuclei are also not well characterized in the awake state. Studying neocortical areas in animals, including with respect to ketamine, has a particularly high degree of translational relevance since human EEG and TMS studies are best suited for monitoring or manipulating neocortex rather than deeper structures. A recent study in humans with treatment-resistant epilepsy, however, used intracranial EEG recordings to identify an amygdala-hippocampus subnetwork, where increased variability in beta-frequency coherence correlated with worsening mood (Kirkby et al. 2018).

Additional lines of inquiry for future studies include comparing awake recordings with the asleep state, in the presence of these drugs. The potential interaction between psychological stress and antidepressants is also not well characterized, electrophysiologically. An understanding of the electrophysiological, circuit-based correlates of individual differences in response to antidepressants is also largely missing at this time. Since human subjects with major depression are in many cases therapeutically resistant to a number of antidepressant drugs, determining neural correlates of individual response in rodents would facilitate translational research toward precision medicine. Future recording studies of antidepressants should address these neglected aspects in the existing literature, especially the poorly understood neocortical effects of these drugs, and thereby elucidate all aspects of the circuitry underlying antidepressant drug response with the high degree of spatiotemporal precision afforded by in vivo electrophysiology, particularly when complemented by optogenetics. Studies of ketamine are especially amenable to electrophysiological investigation in animal models, in that its rapid therapeutic onset mirrors that seen in human subjects (Berman et al. 2000).

An important issue raised by this literature review comprises the extent to which in vivo electrophysiological findings in rodents can be used to improve human pharmacotherapy, including through our understanding of human pharmaconeuroimaging data. We would suggest, for example, that our understanding of ketamine has been reinforced through parallel studies in rodents and humans, where this drug has been shown to boost the power of gamma oscillations across these species (Hakami et al. 2009; Hong et al. 2010; Hunt et al. 2006; Muthukumaraswamy et al. 2015; Shaw et al. 2015). Ketamine, moreover, dose-dependently boosts noradrenaline release in mPFC of freely moving rats (Kubota et al. 1999a, b), which may underlie ketamine’s effects on gamma power, since electrophysiological recordings with the noradrenaline-selective drugs reboxetine (and possibly desipramine) enhance gamma (Hajós et al. 2003). A recent human pharmaconeuroimaging study found that ketamine alters resting state functional connectivity between thalamus and the noradrenergic nucleus, the locus coeruleus (Liebe et al. 2018), consistent with the hypothesis that ketamine augments gamma power via noradrenergic signaling. Since many of the rodent electrophysiological studies of ketamine have focused on modeling schizophrenia, an important new agenda would be to investigate, in greater detail, affective-related behavior while recording with this drug.

In conclusion, much of neuroscience is centered around the idea that action potential generation and synaptic communication are the final common language of nearly all neural functioning, which would include mood, mood regulation, anxiety and motivation. We suggest here that electrophysiologic techniques and understanding have now evolved to the point that they can be applied to problems with less well-defined inputs and outputs than the traditional sensory and motor studies performed by electrophysiologists. In vivo electrophysiology should be used more broadly now to enhance our basic understanding of neuropharmacology, cellular physiology, and both macro- and microcircuitry, with translational relevance for drug discovery and development, while also increasing the clinical effectiveness of antidepressant treatment in major depression and a range of other neuropsychiatric disorders.

## References

[CR1] Adrian ED (1914). The all-or-none principle in nerve. J Physiol.

[CR2] Akhmetshina D, Zakharov A, Vinokurova D, Nasretdinov A, Valeeva G, Khazipov R (2016). The serotonin reuptake inhibitor citalopram suppresses activity in the neonatal rat barrel cortex in vivo. Brain Res Bull.

[CR3] Amirabadi S, Pakdel FG, Shahabi P, Naderi S, Osalou MA, Cankurt U (2014). Microinfusion of bupropion inhibits putative GABAergic neuronal activity of the ventral tegmental area. Basic Clin Neurosci.

[CR4] Andersson JL, Nomikos GG, Marcus M, Hertel P, Mathe JM, Svensson TH (1995). Ritanserin potentiates the stimulatory effects of raclopride on neuronal activity and dopamine release selectively in the mesolimbic dopaminergic system. Naunyn-Schmiedeberg’s Arch Pharmacol.

[CR5] Arborelius L, Nomikos GG, Grillner P, Hertel P, Höök BB, Hacksell U, Svensson TH (1995). 5-HT1A receptor antagonists increase the activity of serotonergic cells in the dorsal raphe nucleus in rats treated acutely or chronically with citalopram. Naunyn-Schmiedeberg’s Arch Pharmacol.

[CR6] Arborelius L, Nomikos GG, Hertel P, Salmi P, Grillner P, Höök BB (1996). The 5-HT1A receptor antagonist (S)-UH-301 augments the increase in extracellular concentrations of 5-HT in the frontal cortex produced by both acute and chronic treatment with citalopram. Naunyn-Schmiedeberg’s Arch Pharmacol.

[CR7] Ashby CR, Edwards E, Wang RY (1992). Action of serotonin in the medial prefrontal cortex: mediation by serotonin, -like receptors. Synapse.

[CR8] Aston-Jones G, Chen S, Zhu Y, Oshinsky ML (2001). A neural circuit for circadian regulation of arousal. Nat Neurosci.

[CR9] Balsara JJ, Jadhav SA, Gaonkar RV, Gaikwad RV, Jadhav JH (2005). Effects of the antidepressant trazodone, a 5-HT 2A/2C receptor antagonist, on dopamine-dependent behaviors in rats. Psychopharmacology.

[CR10] Beique J-C, De Montigny C, Blier P, Debonnel G (1998). Blockade of 5-Hydroxytryptamine and noradrenaline uptake by venlafaxine: a comparative study with paroxetine and desipramine. Br J Pharmacol.

[CR12] Béïque JC, De Montigny C, Blier P, Debonnel G (1999). Venlafaxine: Discrepancy between in vivo 5-HT and NE reuptake blockade and affinity for reuptake sites. Synapse..

[CR13] Béïque JC, De Montigny C, Blier P, Debonnel G (2000). Effects of sustained administration of the serotonin and norepinephrine reuptake inhibitor venlafaxine: I. In vivo electrophysiological studies in the rat. Neuropharmacology.

[CR11] Béïque J-C, Blier P, De Montigny C, Debonnel G (2000). Potentiation by (-)pindolol of the activation of postsynaptic 5-HT(1A) receptors induced by venlafaxine. Neuropsychopharmacology.

[CR14] Belujon P, Grace AA (2014). Restoring mood balance in depression: Ketamine reverses deficit in dopamine-dependent synaptic plasticity. Biol Psychiatry.

[CR15] Belujon P, Jakobowski NL, Dollish HK, Grace AA (2016). Withdrawal from acute amphetamine induces an amygdala-driven attenuation of dopamine neuron activity: reversal by ketamine. Neuropsychopharmacology.

[CR16] Bennett S, Gronier B (2007). Effects of antipsychotic treatments and d-serine supplementation on the electrophysiological activation of midbrain dopamine neurons induced by the noncompetitive NMDA antagonist MK 801. Synapse.

[CR17] Bergqvist PBF, Dong J, Blier P (1999). Effect of atypical antipsychotic drugs on 5-HT 2 receptors in the rat orbito-frontal cortex: an in vivo electrophysiological study. Psychopharmacology.

[CR18] Berman RM, Cappiello A, Anand A, Oren DA, Heninger GR, Charney DS, Krystal JH (2000). Antidepressant effects of ketamine in depressed patients. Biol Psychiatry.

[CR19] Besson A, Haddjeri N, Blier P, De Montig C (2000). Effects of the co-administration of mirtazapine and paroxetine on serotonergic neurotransmission in the rat brain. Eur Neuropsychopharmacol.

[CR20] Bétry C, Pehrson AL, Etiévant A, Ebert B, Sánchez C, Haddjeri N (2013). The rapid recovery of 5-HT cell firing induced by the antidepressant vortioxetine involves 5-HT3 receptor antagonism. Int J Neuropsychopharmacol.

[CR21] Blier P, El Mansari M (2013). Serotonin and beyond: therapeutics for major depression. Philos Trans R Soc B.

[CR22] Blier P, de Montigny C, Chaput Y (1987). Modifications of the serotonin system by antidepressant treatments. J Clin Psychopharmacol.

[CR23] Blier P, de Montigny C, Chaput Y (1990). A role for the serotonin system in the mechanism of action of antidepressant treatments: preclinical evidence. J Clin Psychiatry.

[CR24] Buzsáki G (2010). Neural syntax: cell assemblies, synapsembles, and readers. Neuron.

[CR25] Carlsson A (2006). The neurochemical circuitry of schizophrenia. Pharmacopsychiatry.

[CR26] Cassano GB, Baldini Rossi N, Pini S (2002). Psychopharmacology of anxiety disorders. Dialogues Clin Neurosci.

[CR27] Castañé A, Kargieman L, Celada P, Bortolozzi A, Artigas F (2015). 5-HT2A receptors are involved in cognitive but not antidepressant effects of fluoxetine. Eur Neuropsychopharmacol.

[CR28] Chenu F, Mansari M El, Blier P (2009). Long-term administration of monoamine oxidase inhibitors alters the firing rate and pattern of dopamine neurons in the ventral tegmental area. Int J Neuropsychopharmacol.

[CR29] Chery R, Gurden H, Martin C (2014). Anesthetic regimes modulate the temporal dynamics of local field potential in the mouse olfactory bulb. J Neurophysiol.

[CR30] Chiodo LA, Bunney BS (1983). Typical and atypical neuroleptics: differential effects of chronic administration on the activity of A9 and A10 midbrain dopaminergic neurons’. J Neurosci.

[CR31] Christensen T, Bétry C, Mnie-Filali O, Etievant A, Ebert B, Haddjeri N, Wiborg O (2012). Synergistic antidepressant-like action of gaboxadol and escitalopram. Eur Neuropsychopharmacol.

[CR32] Conley RK, Cumberbatch MJ, Mason GS, Williamson DJ, Harrison T, Locker K (2002). Substance P (neurokinin 1) receptor antagonists enhance dorsal raphe neuronal activity. J Neurosci.

[CR33] Cooper BR, Wang CM, Cox RF, Norton R, Shea V, Ferris RM (1994). Evidence that the acute behavioral and electrophysiological effects of bupropion (Wellbutrin) are mediated by a noradrenergic mechanism. Neuropsychopharmacology.

[CR34] Coyle JT, Tsai G, Goff D (2003). Converging evidence of NMDA receptor hypofunction in the pathophysiology of schizophrenia. Ann N Y Acad Sci..

[CR35] Crespi F (2010). SK channel blocker apamin attenuates the effect of SSRI fluoxetine upon cell firing in dorsal raphe nucleus: A concomitant electrophysiological and electrochemical in vivo study reveals implications for modulating extracellular 5-HT. Brain Res.

[CR206] de Boer T (1996). The pharmacologic profile of mirtazapine. J Clin Psychiatry.

[CR36] Di Giovanni G, De Deurwaerdére P, Di Mascio M, Di Matteo V, Esposito E, Spampinato U (1999). Selective blockade of serotonin-2C/2B receptors enhances mesolimbic and mesostriatal dopaminergic function: a combined in vivo electrophysiological and microdialysis study. Neuroscience.

[CR37] Dimpfel W (2009). Rat electropharmacograms of the flavonoids rutin and quercetin in comparison to those of moclobemide and clinically used reference drugs suggest antidepressive and/or neuroprotective action. Phytomedicine.

[CR38] Dimpfel W, Schombert L, Gericke N (2016). Electropharmacogram of Sceletium tortuosum extract based on spectral local field power in conscious freely moving rats. J Ethnopharmacol.

[CR39] Dremencov E, El Mansari M, Blier P (2007). Distinct electrophysiological effects of paliperidone and risperidone on the firing activity of rat serotonin and norepinephrine neurons. Psychopharmacology.

[CR40] Dremencov E, El Mansari M, Blier P (2007). Noradrenergic augmentation of escitalopram response by risperidone: electrophysiologic studies in the rat brain. Biol Psychiatry.

[CR41] Dremencov E, El Mansari M, Blier P (2009). Effects of sustained serotonin reuptake inhibition on the firing of dopamine neurons in the rat ventral tegmental area. J Psychiatry Neurosci.

[CR42] Dremencov E, Csatlósová K, Durisova B, Moravčíková L, Lacinová L, JezovavPharm DD (2017). Effect of physical exercise and acute escitalopram on the excitability of brain monoamine neurons: in vivo electrophysiological study in rats. Int J Neuropsychopharmacol.

[CR43] Dulawa SC, Hen R (2005). Recent advances in animal models of chronic antidepressant effects: the novelty-induced hypophagia test. Neurosci Biobehav Rev.

[CR45] Dzirasa K, Phillips HW, Sotnikova TD, Salahpour A, Kumar S, Gainetdinov RR (2010). Noradrenergic control of cortico-striato-thalamic and mesolimbic cross-structural synchrony. J Neurosci.

[CR46] Ehlers CL, Kaneko WM, Wall TL, Chaplin RI (1992). Effects of dizocilpine (MK-801) and ethanol on the eeg and event-related potentials (erps) in rats. Neuropharmacology.

[CR47] Einhorn LC, Johansen PA, White FJ (1988). Electrophysiological effects of cocaine in the mesoaccumbens dopamine system: studies in the ventral tegmental area. J Neurosci.

[CR48] El Iskandrani KS, Oosterhof CA, El Mansari M, Blier P (2015). Impact of subanesthetic doses of ketamine on AMPA-mediated responses in rats: an in vivo electrophysiological study on monoaminergic and glutamatergic neurons. J Psychopharmacol.

[CR49] El Mansari M, Blier P (2008). In vivo electrophysiological assessment of the putative antidepressant Wf-516 in the rat raphe dorsalis, locus coeruleus and hippocampus. Naunyn-Schmiedeberg’s Arch Pharmacol.

[CR50] El Mansari M, Sánchez C, Chouvet G, Renaud B, Haddjeri N (2005). Effects of acute and long-term administration of escitalopram and citalopram on serotonin neurotransmission: an in vivo electrophysiological study in rat brain. Neuropsychopharmacology.

[CR51] El Mansari M, Wiborg O, Mnie-Filali O, Benturquia N, Sánchez C, Haddjeri N (2007). Allosteric modulation of the effect of escitalopram, paroxetine and fluoxetine: in vitro and in vivo studies. Int J Neuropsychopharmacol.

[CR52] El Mansari M, Ghanbari R, Janssen S, Blier P (2008). Sustained administration of bupropion alters the neuronal activity of serotonin, norepinephrine but not dopamine neurons in the rat brain. Neuropharmacology.

[CR53] El Mansari M, Guiard BP, Chernoloz O, Ghanbari R, Katz N, Blier P (2010). Relevance of norepinephrine-dopamine interactions in the treatment of major depressive disorder. CNS Neurosci Ther.

[CR54] El Mansari M, Manta S, Oosterhof C, El Iskandrani KS, Chenu F, Shim S, Blier P (2014). Restoration of serotonin neuronal firing following long-term administration of bupropion but not paroxetine in olfactory bulbectomized rats. Int J Neuropsychopharmacol.

[CR55] El Mansari M, Lecours M, Blier P (2015). Effects of acute and sustained administration of vortioxetine on the serotonin system in the hippocampus: electrophysiological studies in the rat brain. Psychopharmacology.

[CR56] Engin E, Stellbrink J, Treit D, Dickson CT (2008). Anxiolytic and antidepressant effects of intracerebroventricularly administered somatostatin: behavioral and neurophysiological evidence. Neuroscience.

[CR57] Engin E, Treit D, Dickson CT (2009). Anxiolytic- and antidepressant-like properties of ketamine in behavioral and neurophysiological animal models. Neuroscience.

[CR58] Espana RA, Scammell TE (2011). Sleep neurobiology from a clinical perspective. Sleep.

[CR59] Fee C, Banasr M, Sibille E (2017). Somatostatin-positive gamma-aminobutyric acid interneuron deficits in depression: cortical microcircuit and therapeutic perspectives. Biol Psychiatry.

[CR60] Fitzgerald PJ (2009). Neuromodulating mice and men: are there functional species differences in neurotransmitter concentration?. Neurosci Biobehav Rev.

[CR61] Fitzgerald PJ, Watson BO (2018). Gamma oscillations as a biomarker for major depression: an emerging topic. Transl Psychiatry.

[CR62] Fitzgerald PJ, Whittle N, Flynn SM, Graybeal C, Pinard CR, Gunduz-Cinar O (2014). Prefrontal single-unit firing associated with deficient extinction in mice. Neurobiol Learn Memory.

[CR63] French ED, Mura A, Wang T (1993). MK-801, phencyclidine (PCP), and PCP-like drugs increase burst firing in rat A10 dopamine neurons: Comparison to competitive NMDA antagonists. Synapse.

[CR64] Frohlich J, Van Horn JD (2014). Reviewing the ketamine model for schizophrenia. J Psychopharmacol.

[CR65] Fujishiro J, Imanishi T, Baba J, Kosaka K (2001). Comparison of noradrenergic and serotonergic antidepressants in reducing immobility time in the tail suspension test. Jpn J Pharmacol.

[CR66] Furuyashiki T, Holland PC, Gallagher M (2008). Rat orbitofrontal cortex separately encodes response and outcome information during performance of goal-directed behavior. J Neurosci.

[CR67] Gallager DW, Aghajanian GK (1975). Effects of chlorimipramine and lysergic acid diethylamide on efflux of precursor-formed 3H-serotonin: correlations with serotonergic impulse flow’. J Pharmacol Exp Ther.

[CR68] Garay RP, Zarate CA, Charpeaud T, Citrome L, Correll CU, Hameg A, Llorca PM (2017). Investigational drugs in recent clinical trials for treatment-resistant depression. Expert Rev Neurother.

[CR69] Ghanbari R, El Mansari M, Blier P (2010). Electrophysiological effects of the co-administration of escitalopram and bupropion on rat serotonin and norepinephrine neurons. J Psychopharmacol.

[CR70] Ghanbari R, Mansari M El, Blier P (2010). Sustained administration of trazodone enhances serotonergic neurotransmission: in vivo electrophysiological study in the rat brain. J Pharmacol Exp Ther.

[CR71] Gire DH, Schoppa NE (2008). Long-term enhancement of synchronized oscillations by adrenergic receptor activation in the olfactory bulb. J Neurophysiol.

[CR72] Gram L (1988). Imipramine: a model substance in pharmacokinetic research. Acta Psychiatr Scand Suppl.

[CR73] Gray CM, Singer W (1989). Stimulus-specific neuronal oscillations in orientation columns of cat visual cortex. Proc Natl Acad Sci.

[CR74] Gronier B (2011). In vivo electrophysiological effects of methylphenidate in the prefrontal cortex: involvement of dopamine D1 and alpha 2 adrenergic receptors. Eur Neuropsychopharmacol.

[CR75] Gronier BS, Rasmussen K (2003). Electrophysiological effects of acute and chronic olanzapine and fluoxetine in the rat prefrontal cortex. Neurosci Lett.

[CR76] Groves SJ, Pitcher TL, Melzer TR, Jordan J, Carter JD, Malhi GS (2018). Brain activation during processing of genuine facial emotion in depression: preliminary findings. J Affect Disord.

[CR77] Guiard BP, El Mansari M, Merali Z, Blier P (2008). Functional interactions between dopamine, serotonin and norepinephrine neurons: an in vivo electrophysiological study in rats with monoaminergic lesions. Int J Neuropsychopharmacol.

[CR78] Guiard BP, Chenu F, Mansari M El, Blier P (2011). Characterization of the electrophysiological properties of triple reuptake inhibitors on monoaminergic neurons. Int J Neuropsychopharmacol.

[CR79] Haddjeri N, Blier P (2000). Effects of sustained (±) pindolol administration on serotonin neurotransmission in rats. J Psychiatry Neurosci.

[CR80] Haddjeri N, Blier P (2001). Sustained blockade of neurokinin-1 receptors enhances serotonin neurotransmission. Biol Psychiatry.

[CR81] Haddjeri N, Blier P (2008). Neurokinin-1 receptor antagonists modulate brain noradrenaline and serotonin interactions. Eur J Pharmacol.

[CR82] Haddjeri N, Blier P, de Montigny C (1995). Noradrenergic modulation of central serotonergic neurotransmission: acute and long-term actions of mirtazapine. Int Clin Psychopharmacol.

[CR83] Haddjeri N, Blier P, De Montigny C, Haddjeri N, Blier P, De Montigny C (1997). Effects of long-term treatment with the a 2 -adrenoceptor antagonist mirtazapine on 5-HT neurotransmission. Naunyn-Schmiedeberg’s Arch Pharmacol.

[CR84] Haddjeri N, Blier P, De Montigny C (1998). Acute and long-term actions of the antidepressant drug mirtazapine on central 5-HT neurotransmission. J Affective Disord.

[CR85] Haddjeri N, De Montigny C, Curet O, Blier P (1998). Effect of the reversible monoamine oxidase A-inhibitor befloxatone on the rat 5-hydroxytryptamine neurotransmission. Eur J Pharmacol.

[CR86] Hadrava V, Blier P, Dennis T, Ortemann C, De Montigny C (1995). Characterization of 5-hydroxytryptamine1A properties of flesinoxan: in vivo electrophysiology and hypothermia study. Neuropharmacology.

[CR87] Hajos M, Gartside SE, Villa AEP, Sharp T (1995). Evidence for a repetitive (burst) firing pattern in a sub-population of 5-hydroxytryptamine neurons in the dorsal and median raphe nuclei of the rat. Neuroscience.

[CR88] Hajos M, Hajos-Korcsok E, Sharp T (1999). Role of the medial prefrontal cortex in 5-HT 1A receptor-induced inhibition of 5-HT neuronal activity in the rat. Br J Pharmacol.

[CR89] Hajós M, Hoffmann WE, Robinson DD, Yu JH, Va Hajó-Korcsok É (2003). Norepinephrine but not serotonin reuptake inhibitors enhance theta and gamma activity of the septo-hippocampal system. Neuropsychopharmacology.

[CR90] Hakami T, Jones NC, Tolmacheva EA, Gaudias J, Chaumont J, Salzberg M (2009). NMDA receptor hypofunction leads to generalized and persistent aberrant γ oscillations independent of hyperlocomotion and the state of consciousness. PLoS One.

[CR91] Hamm JP, Peterka DS, Gogos JA, Yuste R (2017). Altered cortical ensembles in mouse models of schizophrenia. Neuron.

[CR92] Harmer CJ, Bhagwagar Z, Perrett DI, Völlm BA, Cowen PJ, Goodwin GM (2003). Acute SSRI administration affects the processing of social cues in healthy volunteers. Neuropsychopharmacology.

[CR93] Harmer CJ, Shelley NC, Cowen PJ, Goodwin GM (2004). Increased positive versus negative affective perception and memory in healthy volunteers following selective serotonin and norepinephrine reuptake inhibition. Am J Psychiatry.

[CR94] Hodgkin AL, Huxley AF (1952). A quantitative description of membrane current and its application to conduction and excitation in nerve. J Physiol.

[CR95] Homayoun H, Moghaddam B (2007). Fine-tuning of awake prefrontal cortex neurons by clozapine: comparison with haloperidol and N-desmethylclozapine. Biol Psychiat.

[CR96] Hong LE, Summerfelt A, Buchanan RW, O’Donnell P, Thaker GK, Weiler MA, Lahti AC (2010). Gamma and delta neural oscillations and association with clinical symptoms under subanesthetic ketamine. Neuropsychopharmacology.

[CR97] Hubel DH, Wiesel TN (1968). Receptive fields and functional architecture of monkey striate cortex. J Physiol.

[CR98] Hughes ZA, Starr KR, Scott CM, Newson MJ, Sharp T, Watson JM (2007). Simultaneous blockade of 5-HT1A/B receptors and 5-HT transporters results in acute increases in extracellular 5-HT in both rats and guinea pigs: in vivo characterization of the novel 5-HT1A/B receptor antagonist/5-HT transport inhibitor SB-649915-B. Psychopharmacology.

[CR99] Hunt MJ, Raynaud B, Garcia R (2006). Ketamine dose-dependently induces high-frequency oscillations in the nucleus accumbens in freely moving rats. Biol Psychiatry.

[CR100] Hunt MJ, Garcia R, Large CH, Kasicki S (2008). Modulation of high-frequency oscillations associated with NMDA receptor hypofunction in the rodent nucleus accumbens by lamotrigine. Prog Neuro-Psychopharmacol Biol Psychiatry.

[CR101] Hunt MJ, Matulewicz P, Gottesmann C, Kasicki S (2009). State-dependent changes in high-frequency oscillations recorded in the rat nucleus accumbens. Neuroscience.

[CR102] Hunt MJ, Falinska M, Kasicki S (2010). Local injection of MK801 modifies oscillatory activity in the nucleus accumbens in awake rats. J Psychopharmacol.

[CR103] Hunt MJ, Olszewski M, Piasecka J, Whittington MA, Kasicki S (2015). Effects of NMDA receptor antagonists and antipsychotics on high frequency oscillations recorded in the nucleus accumbens of freely moving mice. Psychopharmacology.

[CR104] Hwang E, Mcnally JM, Choi JH, Heck DH, Fontanini A (2013). Reduction in cortical gamma synchrony during depolarized state of slow wave activity in mice. Fort Syst Neurosci.

[CR105] Jadi MP, Behrens MM, Sejnowski TJ (2016). Abnormal gamma oscillations in N-methyl-d-aspartate receptor hypofunction models of schizophrenia. Biol Psychiatry.

[CR106] Jauhar S, Nour MM, Veronese M, Rogdaki M, Bonoldi I, Azis M (2017). A test of the transdiagnostic dopamine hypothesis of psychosis using positron emission tomographic imaging in bipolar affective disorder and schizophrenia. JAMA Psychiatry.

[CR107] Javed A, Van De Kar LD, Gray TS (1998). The 5-HT(1A) and 5-HT(2A/2C) receptor antagonists WAY-100635 and ritanserin do not attenuate d-fenfluramine-induced Fos expression in the brain. Brain Res.

[CR108] Jolas T, Haj-dahmane S, Kidd E, Langlois X, Lanfumey L, Fattaccini C (1994). Central pre-and postsynaptic 5-HTIA receptors in rats treated chronically with a novel antidepressant, cericlamine’. J Pharmacol Exp Ther.

[CR109] Kaeser PS, Regehr WG (2017). The readily releasable pool of synaptic vesicles. Curr Opin Neurobiol.

[CR110] Kalyanasundar B, Perez CI, Luna A, Solorio J, Moreno MG, Elias D (2015). D1 and D2 antagonists reverse the effects of appetite suppressants on weight loss, food intake, locomotion, and rebalance spiking inhibition in the rat NAc shell. J Neurophysiol.

[CR111] Kargieman L, Riga MS, Artigas F, Celada P (2012). Clozapine reverses phencyclidine-induced desynchronization of prefrontal cortex through a 5-HT1A receptor-dependent mechanism. Neuropsychopharmacology.

[CR112] Kasamo K, Blier P, De Montigny C (1996). Blockade of the serotonin and norepinephrine uptake processes by duloxetine: in vitro and in vivo studies in the rat brain. J Pharmacol.

[CR113] Kealy J, Commins S, Lowry JP (2017). The effect of NMDA-R antagonism on simultaneously acquired local field potentials and tissue oxygen levels in the brains of freely-moving rats. Neuropharmacology.

[CR114] Kelland MD, Walters JR (1992). Apomorphine-induced changes in striatal and pallidal neuronal activity are modified by NMDA and muscarinic receptor blockade. Life Sci.

[CR115] Kirkby LA, Luongo FJ, Lee MB, Nahum M, Van Vleet TM, Rao VR (2018). An amygdala-hippocampus subnetwork that encodes variation in human mood. Cell.

[CR116] Kiss T, Hoffmann WE, Hajós M (2011). Delta oscillation and short-term plasticity in the rat medial prefrontal cortex: modelling NMDA hypofunction of schizophrenia. Int J Neuropsychopharmacol.

[CR117] Kiss T, Hoffmann WE, Scott L, Kawabe TT, Milici AJ, Nilsen EA, Hajós M (2011). Role of thalamic projection in NMDA receptor-induced disruption of cortical slow oscillation and short-term plasticity. Front Psychiatry.

[CR118] Kjaerby C, Hovelsø N, Dalby NO, Sotty F (2017). Phencyclidine administration during neurodevelopment alters network activity in prefrontal cortex and hippocampus in adult rats. J Neurophysiol.

[CR119] Kubota T, Anzawa N, Hirota K, Yoshida HM, Kushikata T, Matsuki A (1999). Effects of ketamine and pentobarbital on noradrenaline release from the medial prefrontal cortex in rats. Can J Anaesthesiol.

[CR120] Kubota T, Hirota K, Anzawa N, Yoshida H, Kushikata T, Matsuki A (1999). Physostigmine antagonizes ketamine-induced noradrenaline release from the medial prefrontal cortex in rats. Brain Res.

[CR121] Labonte B, Bambico FR, Gobbi G (2009). Potentiation of excitatory serotonergic responses by MK-801 in the medial prefrontal cortex. Naunyn-Schmiedeberg’s Arch Pharmacol.

[CR122] Lazarewicz MT, Ehrlichman RS, Maxwell CR, Gandal MJ, Finkel LH, Siegel SJ (2010). Ketamine Modulates Theta and Gamma Oscillations. Journal of Cognitive Neuroscience.

[CR123] Lee J, Hudson MR, O’Brien TJ, Nithianantharajah J, Jones NC (2017). Local NMDA receptor hypofunction evokes generalized effects on gamma and high-frequency oscillations and behavior. Neuroscience.

[CR124] Leggio GM, Micale V, Drago F (2008). Increased sensitivity to antidepressants of D3 dopamine receptor-deficient mice in the forced swim test (FST). Eur Neuropsychopharmacol.

[CR125] Leiser SC, Pehrson AL, Robichaud PJ, Sanchez C (2014). Multimodal antidepressant vortioxetine increases frontal cortical oscillations unlike escitalopram and duloxetine—a quantitative EEG study in rats. Br J Pharmacol.

[CR126] Liebe T, Li M, Colic L, Munk MHJ, Sweeney-Reed CM, Woelfer M (2018). Ketamine influences the locus coeruleus norepinephrine network, with a dependency on norepinephrine transporter genotype—a placebo controlled fMRI study. NeuroImage Clin.

[CR127] Linnér L, Endersz H, Ohman D, Bengtsson F, Schalling M, Svensson TH (2001). Reboxetine modulates the firing pattern of dopamine cells in the ventral tegmental area and selectively increases dopamine availability in the prefrontal cortex. J Pharmacol Exp Ther.

[CR128] Linnér L, Arborelius L, Nomikos GG, Bertilsson L, Svensson TH (1999). Locus coeruleus neuronal activity and noradrenaline availability in the frontal cortex of rats chronically treated with imipramine: Effect of α2-adrenoceptor blockade. Biol Psychiatry.

[CR129] Linnér L, Wiker C, Arborelius L, Schalling M, Svensson TH (2004). Selective noradrenaline reuptake inhibition enhances serotonergic neuronal activity and transmitter release in the rat forebrain. J Neural Transm.

[CR130] Lladó-Pelfort L, Assié MB, Newman-Tancredi A, Artigas F, Celada P (2010). Preferential in vivo action of F15599, a novel 5-HT1A receptor agonist, at postsynaptic 5-HT1A receptors. Br J Pharmacol.

[CR131] Lladó-Pelfort L, Troyano-Rodriguez E, Van Den Munkhof HE, Cervera-Ferri A, Jurado N, Núñez-Calvet M (2016). Phencyclidine-induced disruption of oscillatory activity in prefrontal cortex: effects of antipsychotic drugs and receptor ligands. Euro Neuropshyopharmacol.

[CR132] Locher C, Koechlin H, Zion SR, Werner C, Pine DS, Kirsch I (2017). Efficacy and safety of selective serotonin reuptake inhibitors, serotonin-norepinephrine reuptake inhibitors, and placebo for common psychiatric disorders among children and adolescents. JAMA Psychiatry.

[CR133] Lundberg P-A, Oretand L, Engberg G (1985). Inhibition of locus coeruleus neuronal activity by beta-phenylethylamine. Life Sci.

[CR134] Maheshwari A, Marks RL, Yu KM, Noebels JL (2016). Shift in interictal relative gamma power as a novel biomarker for drug response in two mouse models of absence epilepsy. Epilepsia.

[CR135] Marcinkiewcz CA, Mazzone CM, D’Agostino G, Halladay LR, Hardaway JA, Diberto JF (2016). Serotonin engages an anxiety and fear-promoting circuit in the extended amygdala. Nature.

[CR136] Matulewicz P, Kasicki S, Hunt MJ (2010). The effect of dopamine receptor blockade in the rodent nucleus accumbens on local field potential oscillations and motor activity in response to ketamine. Brain Res.

[CR137] Matulewicz P, Orzel-Gryglewska J, Kusmierczak M, Jurkowlaniec E (2014). NMDA-glutamatergic activation of the ventral tegmental area induces hippocampal theta rhythm in anesthetized rats. Brain Res Bull.

[CR138] McEwen BS, Chattarji S, Diamond DM, Jay TM, Reagan LP, Svenningsson P, Fuchs E (2010). The neurobiological properties of tianeptine (Stablon): from monoamine hypothesis to glutamatergic modulation. Mol Psychiatry.

[CR139] Mcmillen BA, Warnack W, German DC, Shore PA (1980). Effects of chronic desipramine treatment on rat brain noradrenergic responses to A-adrenergic drugs *. Eur J Pharmacol.

[CR140] Mendez P, Pazienti A, Szabo G, Bacci A (2012). Direct alteration of a specific inhibitory circuit of the hippocampus by antidepressants. J Neurosci.

[CR141] Mennini T, Mocaer E, Garattini S (1987). Tianeptine, a selective enhancer of serotonin uptake in rat brain. Arch Pharmacol.

[CR142] Milad MR, Quirk GJ (2002). Neurons in medial prefrontal cortex signal memory for fear extinction. Nature.

[CR143] Mnie-Filali O, Mansari M El, Espana A, Sànchez C, Haddjeri N (2006). Allosteric modulation of the effects of the 5-HT reuptake inhibitor escitalopram on the rat hippocampal synaptic plasticity. Neurosci Lett.

[CR144] Moghaddam B, Adams B, Verma A, Daly D (1997). Activation of glutamatergic neurotransmission by ketamine: a novel step in the pathway from NMDA receptor blockade to dopaminergic and cognitive disruptions associated with the prefrontal cortex. J Neurosci.

[CR145] Molina LA, Skelin I, Gruber AJ (2014). Acute NMDA receptor antagonism disrupts synchronization of action potential firing in rat prefrontal cortex. PLoS One.

[CR146] Mongeau R, Blier P, De Montigny C (1993). In vivo electrophysiological evidence for tonic activation by endogenous noradrenaline of a2-adrenoceptors on 5-hydroxytryptamine terminals in the rat hippocampus. Naunyn-Schmiedeberg’s Arch Pharmacol.

[CR147] Monti JM, Monti D (2007). The involvement of dopamine in the modulation of sleep and waking. Sleep Med Rev.

[CR148] Moran RJ, Jones MW, Blockeel AJ, Adams RA, Stephan KE, Friston KJ (2015). Losing control under ketamine: suppressed cortico-hippocampal drive following acute ketamine in rats. Neuropsychopharmacology.

[CR149] Murase S, Nisell M, Grenhoff J, Svensson TH (1992). Decreased sensory responsiveness of noradrenergic neurons in the rat locus coeruleus following phencyclidine or dizocilpine (MK-801): role of NMDA antagonism. Psychopharmacology.

[CR150] Murase S, Mathe JM, Grenhoff J, Svensson TH (1993). Effects of dizocilpine (MK-801) on rat midbrain dopamine cell activity: differential actions on firing pattern related to anatomical localization. J Neural Transm.

[CR151] Muthukumaraswamy SD, Shaw AD, Jackson LE, Hall J, Moran R, Saxena N (2015). Evidence that subanesthetic doses of ketamine cause sustained disruptions of NMDA and AMPA-mediated frontoparietal connectivity in humans. J Neurosci.

[CR152] Nagy D, Stoiljkovic M, Menniti FS, Hajós M (2016). Differential effects of an NR2B NAM and ketamine on synaptic potentiation and gamma synchrony: relevance to rapid-onset antidepressant efficacy. Neuropsychopharmacology.

[CR153] Nasif FJ, Ramírez OA, Cuadra GR, Orsingher OA (2001). Increased neuronal activity in locus coeruleus from adult rats undernourished at perinatal age: its reversal by desipramine. Life Sci.

[CR154] Nicolás MJ, López-Azcárate J, Valencia M, Alegre M, Pérez-Alcázar M, Iriarte J, Artieda J (2011). Ketamine-induced oscillations in the motor circuit of the rat basal Ganglia. PLoS One.

[CR155] Nugent AC, Ballard ED, Gould TD, Park LT, Moaddel R, Brutsche NE, Zarate CA (2018). Ketamine has distinct electrophysiological and behavioral effects in depressed and healthy subjects. Mol Psychiatry.

[CR156] O’Keefe J, Black AH (1977). Single unit and lesion experiments on the sensory inputs to the hippocampal cognitive map. Ciba Found Symp.

[CR157] Olszewski M, Dolowa W, Matulewicz P, Kasicki S, Hunt MJ (2013). NMDA receptor antagonist-enhanced high frequency oscillations: Are they generated broadly or regionally specific?. Eur Neuropsychopharmacol.

[CR158] ou Maati H, Bourcier-Lucas C, Veyssiere J, Kanzari A, Heurteaux C, Borsotto M (2016). The peptidic antidepressant spadin interacts with prefrontal 5-HT4 and mGluR2 receptors in the control of serotonergic function. Brain Struct Funct.

[CR159] Pandipati S, Gire DH, Schoppa NE (2010). Adrenergic receptor-mediated disinhibition of mitral cells triggers long-term enhancement of synchronized oscillations in the olfactory bulb. J Neurophysiol.

[CR160] Parker G, Roy K, Menkes DB, Snowdon J, Boyce P, Grounds D (2000). How long does it take for antidepressant therapies to act?. Aust N Z J Psychiatry.

[CR161] Patton MS, Lodge DJ, Morilak DA, Girotti M (2017). Ketamine corrects stress-induced cognitive dysfunction through JAK2/STAT3 signaling in the orbitofrontal cortex. Neuropsychopharmacology.

[CR162] Peters J, Kalivas PW, Quirk GJ (2009). Extinction circuits for fear and addiction overlap in prefrontal cortex. Learn Memory.

[CR163] Pitkänen M, Sirviö J, Ylinen A, Koivisto E, Riekkinen P (1995). Effects of NMDA receptor modulation on hippocampal type 2 theta activity in rats. Gen Pharmacol.

[CR164] Pratt V, Mcleod H, Rubinstein W, Dean L, Kattman B, Malheiro A (2017) Imipramine therapy and CYP2D6 and CYP2C19 Genotype. Medical Genetics Summaries [Internet]. https://www.ncbi.nlm.nih.gov/pubmed/28520379

[CR165] Puetz TW, Youngstedt SD, Herring MP (2015). Effects of pharmacotherapy on combat-related PTSD, anxiety, and depression: a systematic review and meta-regression analysis. PLoS One.

[CR166] Rasmussen K, Fuller RW, Stockton ME, Perry KW, Swinford RM, Ornstein PL (1991). NMDA receptor antagonists suppress behaviors but not norepinephrine turnover or locus coeruleus unit activity induced by opiate withdrawal. Eur J Pharmacol.

[CR167] Robinson SA, Erickson RL, Browne CA, Lucki I (2017). A role for the mu opioid receptor in the antidepressant effects of buprenorphine. Behav Brain Res.

[CR168] Romer Thomsen K, Whybrow PC, Kringelbach ML (2015). Reconceptualizing anhedonia: novel perspectives on balancing the pleasure networks in the human brain. Front Behav Neurosci.

[CR169] Romero L, Celada P, Martín-Ruiz R, Mourelle M, Delgadillo J, Hervás I, Artigas F (2003). Modulation of serotonergic function in rat brain by VN2222, a serotonin reuptake inhibitor and 5-HT 1A receptor agonist. Neuropsychopharmacology.

[CR170] Rueter LE, De Montigny C, Blier P (1998). Electrophysiological characterization of the effect of long-term duloxetine administration on the rat serotonergic and noradrenergic systems. J Pharmacol Exp The.

[CR171] Ruskin DN, Bergstrom DA, Baek D, Freeman LE, Walters JR (2001). Cocaine or selective block of dopamine transporters influences multisecond oscillations in firing rate in the globus pallidus. Neuropsychopharmacology.

[CR172] Samuels E, Szabadi E (2008). Functional neuroanatomy of the noradrenergic locus coeruleus: its roles in the regulation of arousal and autonomic function part I: principles of functional organisation. Curr Neuropharmacol.

[CR173] Santana N, Troyano-Rodriguez E, Mengod G, Celada P, Artigas F (2011). Activation of thalamocortical networks by the N-methyl-d-aspartate receptor antagonist phencyclidine: reversal by clozapine. Biol Psychiatry.

[CR174] Schilström B, Konradsson-Geuken Å, Ivanov V, Gertow J, Feltmann K, Marcus MM (2011). Effects of S-citalopram, citalopram, and R-citalopram on the firing patterns of dopamine neurons in the ventral tegmental area, N-methyl-d-aspartate receptor-mediated transmission in the medial prefrontal cortex and cognitive function in the rat. Synapse.

[CR175] Scuvée-Moreau JJ, Svensson TH (1982). Sensitivity in vivo of central a 2-and opiate receptors after chronic treatment with various antidepressants. J Neural Transm.

[CR176] Seager MA, Huff KD, Barth VN, Phebus LA, Rasmussen K (2004). Fluoxetine administration potentiates the effect of olanzapine on locus coeruleus neuronal activity. Biol Psychiatry.

[CR177] Seager MA, Barth VN, Phebus LA, Rasmussen K (2005). Chronic coadministration of olanzapine and fluoxetine activates locus coeruleus neurons in rats: implications for bipolar disorder. Psychopharmacology.

[CR178] Sekine Y, Suzuki K, Ramachandran PV, Blackburn TP, Ashby CR (2007). Acute and repeated administration of fluoxetine, citalopram, and paroxetine significantly alters the activity of midbrain dopamine neurons in rats: an in vivo electrophysiological study. Synapse.

[CR179] Sharma AV, Wolansky T, Dickson CT (2010). A comparison of sleeplike slow oscillations in the hippocampus under ketamine and urethane anesthesia. J Neurophysiol.

[CR180] Shaw AD, Saxena N, Jackson LE, Hall JE, Singh KD, Muthukumaraswamy SD (2015). Ketamine amplifies induced gamma frequency oscillations in the human cerebral cortex. Eur Neuropsychopharmacol.

[CR181] Shi W-X, Pun C-L, Zhang X-X, Jones MD, Bunney BS (2000). Dual effects of D-amphetamine on dopamine neurons mediated by dopamine and nondopamine receptors. J Neurosci.

[CR182] Shim S, Mansari M El, Blier P (2013). Modulation of the antidepressant-like effects of sustained administration of carisbamate and lamotrigine on monoaminergic systems: electrophysiological studies in the rat brain. J Pharmacol Exp Ther.

[CR183] Shirokawa T, Ishida Y, Isobe K-I (2003). Age-related changes in the release and uptake activity of presynaptic axon terminals of rat locus coeruleus neurons. Neurosci Lett.

[CR184] Skoblenick KJ, Womelsdorf T, Everling S (2016). Ketamine alters outcome-related local field potentials in monkey prefrontal cortex. Cereb Cortex.

[CR185] Slovik M, Rosin B, Moshel S, Mitelman R, Schechtman E, Eitan R (2017). Ketamine induced converged synchronous gamma oscillations in the cortico-basal ganglia network of nonhuman primates. J Neurophysiol.

[CR186] Sodero AO, Valdomero A, Cuadra GR, Ramírez OA, Orsingher OA (2004). Locus coeruleus activity in perinatally protein-deprived rats: effects of fluoxetine administration. Eur J Pharmacol.

[CR187] Sotty F, Folgering JHA, Brennum LT, Hogg S, Mørk A, Hertel P, Cremers TIFH (2009). Relevance of dorsal raphe nucleus firing in serotonin 5-HT2C receptor blockade-induced augmentation of SSRIs effects. Neuropharmacology.

[CR188] Sprouse J, Braselton J, Reynolds L (2000). 5-HT 1A agonist potential of pindolol: electrophysiologic studies in the dorsal raphe nucleus and hippocampus. Biol Psychiat.

[CR189] Stalnaker TA, Cooch NK, Schoenbaum G (2015). What the orbitofrontal cortex does not do. Nat Neurosci.

[CR190] Steinfels GF, Tam SW, Cook L, Whittington MA, Kasicki S (1989). Electrophysiological effects of selective sigma-receptor agonists, antagonists, and the selective phencyclidine receptor agonist MK-801 on midbrain dopamine neurons. Neuropsychopharmacology.

[CR191] Strandgarden K, Gunnarsson P (1994). Metabolism of lofepramine and imipramine in liver microsomes from rat and man. Xenobiotica.

[CR192] Sullivan EM, Timi P, Hong LE, O’Donnell P (2015). Reverse translation of clinical electrophysiological biomarkers in behaving rodents under acute and chronic NMDA receptor antagonism. Neuropsychopharmacology.

[CR193] Svensson TH, Usdin T (1978). Feedback inhibition of brain noradrenaline neurons by tricyclic antidepressants: α-receptor mediation. Science.

[CR194] Szegedi V, Juhász G, Zhang X, Barkóczi B, Qi H, Madeira A (2011). Tianeptine potentiates AMPA receptors by activating CaMKII and PKA via the p38, p42/44 MAPK and JNK pathways. Neurochem Int.

[CR195] Touiki K, Rat P, Molimard R, Chait A, De Beaurepaire R (2005). Harmane inhibits serotonergic dorsal raphe neurons in the rat. Psychopharmacology.

[CR196] Troyano-Rodriguez E, Lladó-Pelfort L, Santana N, Teruel-Martí V, Celada P, Artigas F (2014). Phencyclidine inhibits the activity of thalamic reticular gamma-aminobutyric acidergic neurons in rat brain. Biol Psychiat.

[CR197] Ugedo L, Grenhoff J, Svensson TH (1989). Ritanserin, a 5-HT2 receptor antagonist, activates midbrain dopamine neurons by blocking serotonergic inhibition. Psychopharmacology.

[CR198] Van der Maelen CP, Braselton JP (1990). Acute administration of the antidepressant trazodone increases noradrenergic locus coeruleus neuronal firing in rats. Arch Int Pharmacodyn Ther.

[CR199] Vazey EM, Aston-Jones G (2014). Designer receptor manipulations reveal a role of the locus coeruleus noradrenergic system in isoflurane general anesthesia. Proc Natl Acad Sci.

[CR200] Vidal-Gonzalez I, Vidal-Gonzalez B, Rauch SL, Quirk GJ (2006). Microstimulation reveals opposing influences of prelimbic and infralimbic cortex on the expression of conditioned fear. Learn Memory.

[CR201] Watson BO, Ding M, Buzsaki G (2018). Temporal coupling of field potentials and action potentials in the neocortex. Eur J Neurosci.

[CR202] Weisstaub NV, Zhou M, Lira A, Lambe E, González-Maeso J, Hornung JP, Sibille E, Underwood M, Itohara S, Dauer WT, Ansorge MS, Morelli E, Mann JJ, Toth M, Aghajanian G, Sealfon SC, Hen RGJ (2006). Cortical 5-HT2A receptor signaling modulates anxiety-like behaviors in mice. Science.

[CR203] Wood J, Kim Y, Moghaddam B (2012). Disruption of prefrontal cortex large scale neuronal activity by different classes of psychotomimetic drugs. J Neurosci.

[CR204] Zhang J, Chiodo LA, Freeman AS (1992). Electrophysiological effects of MK-801 on rat nigrostriatal and mesoaccumbal dopaminergic neurons. Brain Res.

[CR205] Zhang H, Harris L, Split M, Troiani V, Olson IR (2016). Anhedonia and individual differences in orbitofrontal cortex sulcogyral morphology. Hum Brain Mapp.

